# Effects of Whole Grain, Fish and Bilberries on Serum Metabolic Profile and Lipid Transfer Protein Activities: A Randomized Trial (Sysdimet)

**DOI:** 10.1371/journal.pone.0090352

**Published:** 2014-02-28

**Authors:** Maria Lankinen, Marjukka Kolehmainen, Tiina Jääskeläinen, Jussi Paananen, Laura Joukamo, Antti J. Kangas, Pasi Soininen, Kaisa Poutanen, Hannu Mykkänen, Helena Gylling, Matej Orešič, Matti Jauhiainen, Mika Ala-Korpela, Matti Uusitupa, Ursula Schwab

**Affiliations:** 1 Department of Clinical Nutrition, Institute of Public Health and Clinical Nutrition, University of Eastern Finland, Kuopio Campus, Kuopio, Finland; 2 VTT, Technical Research Centre of Finland, Kuopio, Finland; 3 Computational Medicine, Institute of Health Sciences, Faculty of Medicine, University of Oulu, Oulu, Finland; 4 NMR Metabolomics Laboratory, School of Pharmacy, University of Eastern Finland, Kuopio, Finland; 5 VTT, Technical Research Centre of Finland, Espoo, Finland; 6 Division of Internal Medicine, Department of Medicine, University of Helsinki, Helsinki, Finland; 7 National Institute for Health and Welfare, Public Health Genomics Research Unit, Biomedicum, Helsinki, Finland; 8 Computational Medicine, School of Social and Community Medicine and the Medical Research Council Integrative Epidemiology Unit, University of Bristol, Bristol, United Kingdom; 9 Oulu University Hospital, Oulu, Finland; 10 Research Unit, Kuopio University Hospital, Kuopio, Finland; 11 Institute of Clinical Medicine, Internal Medicine, Kuopio University Hospital, Kuopio, Finland; Bielefeld Evangelical Hospital, Germany

## Abstract

**Objective:**

We studied the combined effects of wholegrain, fish and bilberries on serum metabolic profile and lipid transfer protein activities in subjects with the metabolic syndrome.

**Methods:**

Altogether 131 subjects (40–70 y, BMI 26–39 kg/m^2^) with impaired glucose metabolism and features of the metabolic syndrome were randomized into three groups with 12-week periods according to a parallel study design. They consumed either: a) wholegrain and low postprandial insulin response grain products, fatty fish 3 times a week, and bilberries 3 portions per day (HealthyDiet), b) wholegrain and low postprandial insulin response grain products (WGED), or c) refined wheat breads as cereal products (Control). Altogether 106 subjects completed the study. Serum metabolic profile was studied using an NMR-based platform providing information on lipoprotein subclasses and lipids as well as low-molecular-weight metabolites.

**Results:**

There were no significant differences in clinical characteristics between the groups at baseline or at the end of the intervention. Mixed model analyses revealed significant changes in lipid metabolites in the HealthyDiet group during the intervention compared to the Control group. All changes reflected increased polyunsaturation in plasma fatty acids, especially in n-3 PUFAs, while n-6 and n-7 fatty acids decreased. According to tertiles of changes in fish intake, a greater increase of fish intake was associated with increased concentration of large HDL particles, larger average diameter of HDL particles, and increased concentrations of large HDL lipid components, even though total levels of HDL cholesterol remained stable.

**Conclusions:**

The results suggest that consumption of diet rich in whole grain, bilberries and especially fatty fish causes changes in HDL particles shifting their subclass distribution toward larger particles. These changes may be related to known protective functions of HDL such as reverse cholesterol transport and could partly explain the known protective effects of fish consumption against atherosclerosis.

**Trial Registration:**

The study was registered at ClinicalTrials.gov NCT00573781.

## Introduction

Wholegrain, fish, and polyphenol rich foods, such as berries, are known to have beneficial effects on human health [1]–[3]. These food items are also part of the Nordic diet, which has recently raised a great interest. In an observational study, healthy Nordic food pattern has been shown to be associated with reduced mortality in adults [4]. In intervention studies improvements in cardiovascular and diabetic risk markers, especially low-grade inflammation, in high risk individuals have been reported [5]–[7]. Overall, nutritional studies are shifting increasingly their focus from studying single nutrients or foods on the exploration of the whole dietary pattern. The Mediterranean diet is probably the best-known dietary pattern and has long been related to improved health and prevention of e.g. cardiovascular diseases (CVD) and type 2 diabetes [8]–[10].

Epidemiological evidence is strong concerning the beneficial effects of consumption of fish on the prevention of coronary heart disease (CHD) progression and mortality [11], [12]. The hypotriglyceridemic effect of fish oil is also well documented, but the evidence has been obtained from trials using fish oil supplements and in larger amounts than currently recommended [13], [14]. Instead, there is no evidence of a secondary preventive effect of n-3 fatty acid supplements against overall cardiovascular events among patients with a history of cardiovascular disease [15]. The effect on glucose and insulin metabolism has been inconsistent, but possible adverse effects on glucose metabolism related to especially higher doses may be compensated by improved serum triglyceride concentration in diabetic subjects [14], [16], [17]. Recent controlled experimental studies have focused on the effects of supplemental long chain n-3 polyunsaturated fatty acids rather than the effects of dietary fish.

The use of metabolomics in nutrition-related research has made a significant impact on assessing novel biomarkers of dietary intake as well as analyses of intervention studies [18]. Mass spectrometry and proton nuclear magnetic resonance (NMR) spectroscopy are the two key experimental technologies in the field. NMR spectroscopy could provide information on lipoprotein subclass distribution and lipoprotein particle concentrations, low-molecular-weight metabolites as well as detailed molecular information on serum lipids [19], [20]. It is suggested that besides the measurement of standard lipids, including total cholesterol, LDL cholesterol and HDL cholesterol concentrations, the measurement of specific HDL subclasses might help in evaluation of the risk of cardiovascular events [21]. Increased concentration of large HDL particles has inverse association with the risk of coronary artery disease, while small HDL particles appear to be associated with increased risk [21], [22]. Lipid transfer proteins phospholipid transfer protein (PLTP) and cholesterol ester transfer protein (CETP) are among factors which modify HDL composition and also affect efficiency of HDL-mediated reverse cholesterol transport [23]–[25]. HDL associated enzyme paraoxonase 1 (PON1) also is an important factor regulating anti-oxidative processes of lipoprotein particles, mainly LDL and HDL [26].

Few studies so far examined cross-sectional association between nutrients and lipoprotein subclasses suggest that alcohol, disaccharides, and n-3 PUFAs are associated with serum lipoprotein subclass distribution [27]–[30]. Controlled trials have focused on n-3 PUFAs, mainly using supplements. Most studies investigating the effects of n-3 PUFAs or fatty fish on HDL particle subspecies have reported increases in large HDL particles and/or decreases of small HDL particles [31]. In our pilot study, fatty fish intake at least 4 times per week increased HDL particle size [32]. Considering whole dietary patterns, positive associations have been reported between a high-fat, high-sucrose and low fiber dietary pattern (junk food) and concentrations of serum triglycerides (Tg) and small dense HDL particles, larger Tg-enriched VLDL particles and small dense LDL particles [33].

We have previously shown that a diet rich in whole grain and low insulin response grain products, fatty fish and berries alter plasma lipidomic profile and improves glucose metabolism and markers of endothelial function and inflammation [6], [34]. In the present secondary analyses of the same trial, we take the advantage of the comprehensive information provided by the new serum NMR-based metabolomics platform and examine the combined effects of these food items on the systemic metabolic profiles and lipid transfer protein activities in subjects with the metabolic syndrome. Furthermore, we study the associations between the changes in fish intake and changes in variables related to large HDL particles, and cross-sectional correlations between the lipid transfer protein activities and variables related to large HDL particles, and between average diameter of HDL and LDL particles and serum Tg.

## Materials and Methods

### Ethics statement

The participants volunteered to the study and gave their written informed consent. The study plan was approved by the Research Ethics Committee, Hospital District of Northern Savo. The intervention was performed in accordance of Helsinki Declaration.

The study was registered at ClinicalTrials.gov NCT00573781. The trial was registered after participants’ recruitment began. The registration was delayed, because of the fact that the procedure of trial registration had just began to be a common practice at that time (2007), and we were not aware of the details related to the time limits. However, we did not start recruitment before the study plan was approved by the Research Ethics Committee. The trial was registered before the intervention began.

The protocol for this trial and completed CONSORT checklist are available as supporting information; See Checklist S1 and Protocol S1. The data is available from authors.

### Subjects

**Figure 1:**
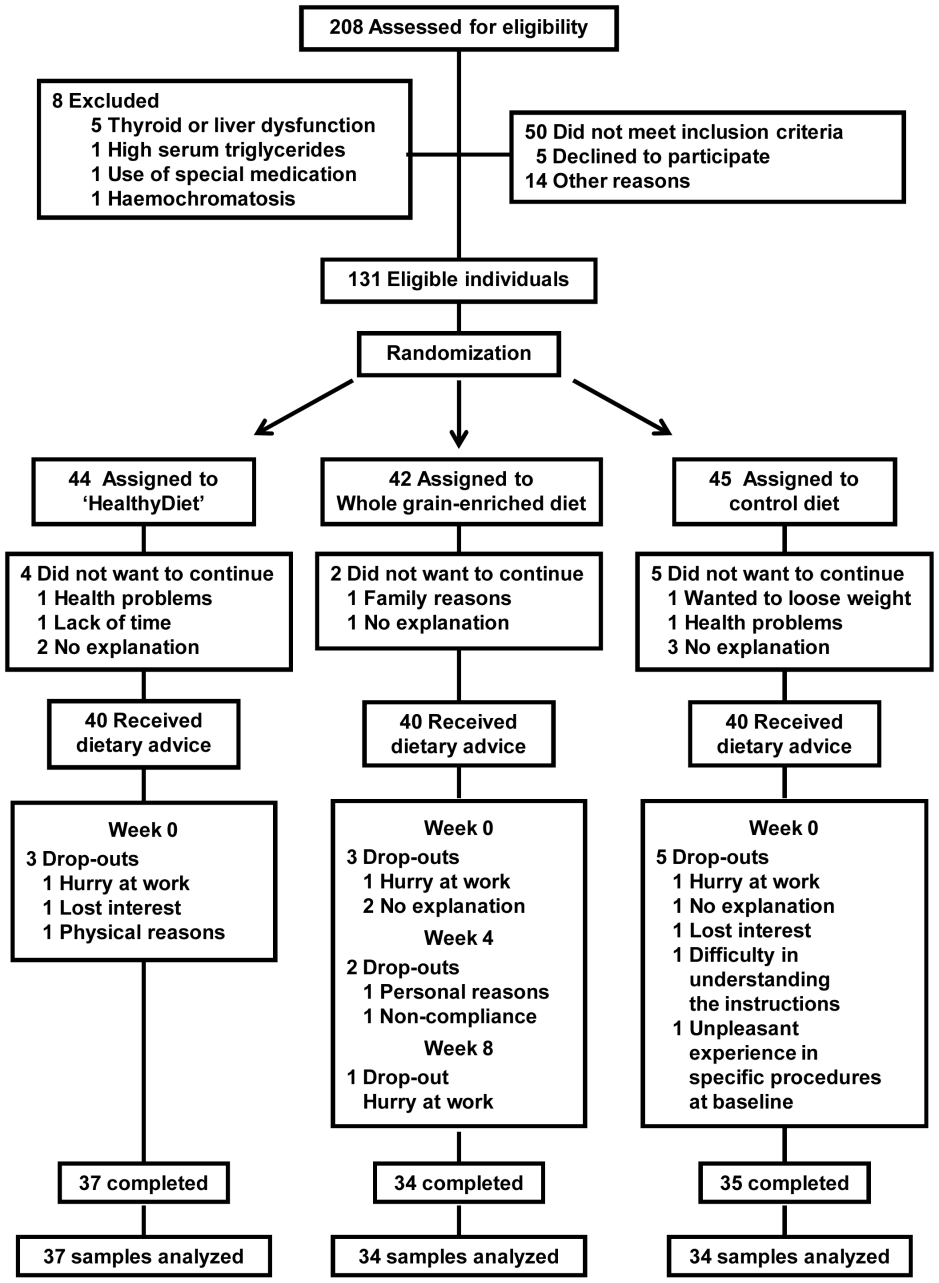
Flow chart of the study. Adapted from De Mello et al [6].

Altogether 131free-living subjects of Caucasian origin were recruited into a 12-week parallel controlled dietary intervention trial via advertisements in the local newspaper in Kuopio area. The trial was conducted at the Department of Clinical Nutrition, University of Eastern Finland (Kuopio, Finland). Recruitment and screening were done during October 2007 - November 2008, and intervention periods were carried out during January 2008 - June 2009. The inclusion criteria were age 40–70 years, impaired glucose metabolism (fP-gluc 5.6–6.9 mmol/l or in OGTT 2 hour P-gluc 7.8–11.0 mmol/l) and two of the following: BMI 26–39 kg/m^2^, waist circumference >102 cm in men and >88 cm in women, serum Tg >1.7 mmol/l, HDL <1.0 mmol/l in men and <1.3 mmol/l in women or blood pressure ≥130 or ≥85 mmHg (NCEP Adult Treatment Panel III, 2001). After a baseline period of two weeks with unchanged diet and lifestyle habits, the subjects were randomized by study nurse into three groups; HealthyDiet, Wholegrain Enriched Diet (WGED) or Control. The randomization was conducted based on a randomization table by matching the subjects according to gender and medians of age, fP-gluc and BMI of the study population at screening so that from every division of randomizing factors (gender->age->BMI->fP-glucose) it was possible to end up to any of the three study groups. The matching produced equal amounts of certain strata classes among the groups. Altogether 106 subjects completed the study (Figure 1). Serum samples for a high-throughput ^1^H-NMR analyses were available from 105 participants. The drop outs did not differ in clinical characteristics except they were younger than the completers (55±8 vs. 59±7 years, mean ± SD, p =  0.023).

Baseline characteristics of the subjects are presented in Table 1. Of all subjects, 86% had at least one medication. Subjects were asked to continue their medication unchanged throughout the study. Of all subjects, 27% used statins. The use of statins or other medication did not differ between the groups. Detailed information about the medication of the participants has been reported earlier [34].

**Table 1:** Clinical characteristics at baseline and at the end of the intervention period (mean ± SD).

	HealthyDiet n = 37				WGED n = 34				Control n = 35	
	0 wk	12 wk	p 1	FDR p 1	0 wk	12 wk	p 1	FDR p 1	0 wk	12 wk
**Body mass index (kg/m^2^)**	31.1±3.6	30.9±3.4	0.860	0.966	31.4±3.4	31.4±3.4	0.171	0.675	31.0±3.6	31.2±3.7
**Waist circumference (cm)**	105.6±9.5	105.0±9.1	0.008	0.091	106.3±11.1	105.9±11.4	0.034	0.406	105.8±10.0	106.3±9.8
**Systolic blood pressure (mmHg)**	137±13	132±13	0.453	0.833	135±16	133±15	0.453	0.805	139±12	135±13
**Diastolic blood pressure (mmHg)**	89±7	86±7	0.596	0.833	86±8	84±7	0.592	0.833	88±7	86±6
**Serum total cholesterol (mmol/l)**	5.1±0.9	5.2±0.8	0.396	0.793	5.1±1.0	5.3±1.1	0.160	0.675	5.4±1.0	5.4±1.0
**LDL cholesterol (mmol/l)**	3.1±0.7	3.1±0.7	0.804	0.953	3.2±0.8	3.2±0.9	0.334	0.712	3.4±0.8	3.4±0.8
**HDL cholesterol (mmol/l)**	1.3±0.3	1.3±0.3	0.966	0.966	1.2±0.4	1.2±0.4	0.919	0.966	1.3±0.3	1.3±0.3
**Serum triglycerides (mmol/l)**	1.6±0.6	1.7±0.8	0.250	0.712	1.5±0.8	1.7±1.0	0.951	0.966	1.5±0.8	1.6±0.7
**Serum ApoA-1 (g/l)**	1.5±0.2	1.5±0.2	0.673	0.847	1.4±0.3	1.4±0.3	0.706	0.847	1.4±0.2	1.4±0.2
**Serum ApoB100 (g/l)**	1.1±0.2	1.1±0.2	0.867	0.867	1.1±0.3	1.1±0.3	0.487	0.847	1.1±0.3	1.1±0.3
**ApoB100/Apo A-1**	0.72±0.17	0.71±0.16	0.570	0.847	0.79±0.21	0.77±0.19	0.671	0.847	0.81±0.22	0.79±0.23
**Fasting plasma glucose (mmol/l)**	6.1±0.5	6.1±0.5	0.728	0.932	6.1±0.4	6.1±0.5	0.506	0.833	6.2±0.5	6.2±0.5
**2-h plasma glucose (mmol/l)**	6.7±1.7	6.0±1.8	0.099	0.530	6.6±1.6	6.1±1.9	0.211	0.675	6.8±1.9	6.7±2.2
**Serum insulin (mU/l)**	11.7±5.9	12.5±6.3	0.624	0.833	12.0±6.2	13.7±8.0	0.316	0.712	13.0±6.7	13.2±6.3
**PLTP activity (nmol/ml/h)**	7288±1686	7249±1560	0.750	0.947	7489±1958	7339±1609	0.947	0.947	6985±1353	6921±1522
**CETP activity (nmol/ml/h)**	25.8±6.9	25.4±6.9	0.607	0.947	24.9±6.3	25.1±6.0	0.719	0.947	25.9±7.4	25.8±6.9
**PON-1 activity (µmol/min)**	26.6±24.0	25.3±21.7	0.890	0.947	29.0±24.4	27.8±23.4	0.881	0.947	21.3±16.0	20.4±14.1

1 Based on a mixed model comparison, where the interaction of time and group compared to Controls (time*group) is analyzed.

### Diets

In the HealthyDiet group (n = 37), the subjects replaced their habitual grain products with whole grain breads and a bread with low postprandial insulin response as previously reported [34]. In brief, these products covered 20–25% of total energy intake and were delivered to the subjects. The fiber contents of the breads were 6.9% (endosperm rye bread), 6.4% (wholegrain wheat bread) and 10–14% (commercial whole grain rye breads). One portion of habitually used grain product, e.g. a slice of low fiber wheat bread, was allowed daily to increase compliance. Pasta with the fiber content of 6% was delivered as well and it was instructed to be consumed at the dose equal to 3.5 deciliter of uncooked pasta per week. The subjects were instructed to eat fatty fish (á 100–150 g) three times a week. Fish was reimbursed to the subjects. Bilberries were instructed to be consumed three portions per day (one portion corresponding to 100 g of fresh bilberries). Bilberries were delivered as whole frozen berries, frozen berry pure and berry powder made of whole berries without added sugar. Other berries were allowed to be consumed 3–4 portions (á ≤1 dl) per week.

In the WGED group (n =  34) the subjects consumed the same grain products as in the HealthyDiet group. In addition, the subjects could voluntarily have a wholegrain oat snack (8–8.5% dietary fiber) 1 portion per day. Eating habits regarding fish and berries were asked to be kept unchanged.

In the Control group (n = 35) the subjects replaced their habitually used grain products with refined wheat breads (dietary fiber 3–4.3 g/100 g) and other low fiber products (<6% dietary fiber) which were delivered to the subjects. Maximum 1–2 portions of rye products were allowed per day. Bilberries were not allowed and other berries were allowed maximum 3–4 portions per week. A fish meal was allowed not more than once a week.

Other eating and lifestyle habits were asked to be kept unchanged. Alcohol use, physical exercise and medications were asked at screening, in the beginning and at the end of the study using a structured questionnaire. Dietary intake was monitored by 4-d food records which were kept during the run in period and three times during the intervention period at the 3^rd^, 7^th^ and 11^th^ week of the intervention. The recording dates were given by the study personnel and were either from Wednesday to Saturday or from Sunday to Wednesday. Nutrica® dietary intake analysis software (v. 2.5) was used for calculations. It is based on Finnish food composition analyses and international food composition tables. In addition to the food records, the subjects in all groups kept consumption records regarding the intake of fish, grain products and berries throughout the intervention to ensure compliance. These records were checked each time the subjects reported to the study center (every 1 to 2 weeks). Besides the daily records, the intake of fish was calculated manually based on 4-d food records.

### Biochemical analyses

Fasting samples for biochemical analyses were drawn from an antecubital vein. Methods for an oral glucose tolerance test (OGTT), plasma glucose, serum insulin, serum cholesterol and serum Apo A-1 and ApoB100 analyses are described in supplementary text (Method S1).

### Serum NMR Metabolomics

We applied proton NMR spectroscopy to determine the serum metabolome via 3 different molecular windows [19], [20]. These windows provide information on 14 lipoprotein subclasses and lipoprotein particle concentrations (LIPO), low-molecular-weight metabolites such as amino acids, 3-hydroxybutyrate, and creatinine (LMWM), and detailed molecular information on serum lipids including free and esterified cholesterol, sphingomyelin, saturation degree and ω-3 fatty acids (LIPID). The methodology provides information on >100 primary metabolic measures and multiple derived variables with clear biochemical interpretation and significance [20], [35]. The LIPO+LMWM data are run from native serum samples in the same experiment and the LIPID from the lipid extracts afterwards. The experimental protocol including sample preparation and NMR spectroscopy are described in detail in Inouye et al. [36]. The definition of the lipoprotein subclasses is as follows: chylomicrons and largest VLDL particles (average particle diameter at least 75 nm); five different VLDL subclasses: very large VLDL (average particle diameter of 64.0 nm), large VLDL (53.6 nm), medium VLDL (44.5 nm), small VLDL (36.8 nm), and very small VLDL (31.3 nm); intermediate-density lipoprotein (IDL) (28.6 nm); three LDL subclasses: large LDL (25.5 nm), medium LDL (23.0 nm), and small LDL (18.7 nm); and four HDL subclasses: very large HDL (14.3 nm), large HDL (12.1 nm), medium HDL (10.9 nm), and small HDL (8.7 nm). The following components of the lipoprotein particles were quantified: phospholipids, Tg, cholesterol, free cholesterol, and cholesterol esters. Due to resolution and concentration issues all of these components are not available for every subclass [37]. The complete list of quantified metabolic measures is given in Supplemental Table S1. This quantitative platform has recently been applied in various large-scale epidemiological and genetic studies [35]–[40]. Serum samples for NMR spectroscopy were available at baseline and at the end of the intervention from 105 participants. Thus 210 samples were originally processed, but 12 samples were lost due to experimental problems.

### PLTP, CETP and PON1

Radiometric methods were used to analyze CETP [41] and PLTP activities [42]. Serum PON1 activity was measured using paraoxon as a substrate [43].

### Statistical analyses

Based on primary aim of seeing changes in glucose metabolism, the power calculations were calculated based on fasting glucose. By using test significance α-level 0.05, 80% power, SD 0.4665 for variability, aim to see 5% difference between the groups in fasting glucose and power analysis method for two-sample t-test, the appropriate sample size was 37 per group. Statistical analyses were performed using the IBM SPSS statistical software (v. 19, SPSS Inc., Chicago, IL), R Project for Statistical Computing version 2.7.2 [44] and nlme R-package version 3.1–96 [45]. Linear mixed-effect models were used to analyse group differences in the baseline and during the intervention. Quantitative dependent variables were transformed to base-10 logarithmic scale to account for non-normal distributions. Selected confounding phenotypes (gender, age, fasting glucose, BMI, cholesterol lowering medication, diuretic medication and smoking, were included in the models as covariates when studying changes in the NMR-based metabolic measures and subject identifiers were included as a grouping random effect. Interaction term between group and intervention time-point (before or after intervention) was used to examine group related changes during the intervention. Control group was used as a reference group when comparing group differences. Benjamini–Hochberg false discovery rate (FDR) was used to adjust the metabolomics measures and dietary intake results for multiple comparisons [46]. Adjustment was done by one treatment effect at a time and in clinically relevant sets. FDR-adjusted p-value ≤ 0.05 was considered as statistically significant. All these analyses are secondary though aimed to be done in the original study protocol. The primary aim of the study was to examine effects on glucose metabolism [6], [34].

Associations between the changes in fish intake and changes in variables related to large HDL particles were tested using analyses of covariance (ANCOVA). Intervention group, age, gender, BMI and fasting glucose were included into models. Analyses were performed using standardized variables. Differences between the fish intake tertiles were tested using one-way ANOVA and Bonferroni post hoc comparisons. Baseline associations were studied applying linear regression model including age, gender, BMI and fasting glucose into models.

## Results

### Clinical characteristics

The gender and age distributions of the study subjects were similar in all three groups [6], [34]. The average age was 58±7, 58±8 and 59±7 years, and the gender distribution (male/female) was 17/20, 17/17 and 18/17 in the HealthyDiet, WGED and Control group, respectively. Table 1 shows the clinical characteristics at baseline and at the end of the intervention. There were no significant differences in the characteristics between the groups at baseline or at the end of the intervention.

### Dietary intake

In the HealthyDiet group, average fish consumption was 3.3 fish meals per week (67 g per day). Mostly used fish species were salmon, rainbow trout, vendace and Baltic herring. The average consumption of delivered bilberries and breads was 3.2 portions and 7.7 portions per day, respectively. The average consumption of the delivered breads was 7.9 portions in the WGED group and 6.8 portions in the Control group per day. The average fish intake was 42 g and 16 g per day in the WGED and in the Control group, respectively.

At baseline, the intake of dietary fiber was higher in the HealthyDiet group than in the WGED and Control groups (FDR p = 0.013). There were no other differences in the dietary intake at baseline. Dietary intake is presented in Table 2. In the HealthyDiet group, eicosapentaenoic acid (EPA), docosahexaenoic acid (DHA) and α-linolenic acid intakes increased while in the Control group the intake of PUFA, EPA and DHA decreased. The decrease of SFA intake in the Healthy Diet group compared to Control group was nearly significant (FDR p =  0.09). In the WGED group, the intake of total fat decreased, but there was no change in the quality of dietary fat. Fiber intake increased in both HealthyDiet and WGED groups whereas it decreased in the Control group.

**Table 2:** Daily dietary intakes at baseline and during the intervention (mean ± SD).

	HealthyDiet n = 36				WGED n = 34				Control n = 35	
	Baseline^1^	Intervention^2^	p^3^	FDR p^3^	Baseline^1^	Intervention^2^	p^3^	FDR p^3^	Baseline^1^	Intervention^2^
Energy (kJ)	8386±2196	9146±2206	0.053	0.121	6995±2373	7654±2395	0.119	0.204	7282±2011	8533±1693
Total fat (E%)	32.5±6.2	30.5±4.6	0.064	0.129	33.6±5.2	31.1±6.3	0.012	0.043	31.3±5.3	31.9±5.9
SAFA (E%)	12.0±3.1	10.8±2.5	0.034	0.090	12.3±2.4	11.1±2.8	0.024	0.070	12.0±2.6	12.1±2.7
MUFA (E%)	10.6±2.6	9.5±1.9	0.171	0.261	11.3±2.1	9.8±2.7	0.020	0.065	10.2±2.4	9.9±2.6
PUFA (E%)	5.6±1.5	5.4±1.2	0.011	0.043	6.0±1.6	5.0±1.5	0.902	0.962	5.6±1.5	4.6±1.2
Linoleic acid (E%)	3.78±1.2	3.32±1.02	0.310	0.381	4.27±1.39	3.34±1.32	0.434	0.515	3.83±1.09	3.18±0.85
α-linolenic acid (E%)	0.84±0.29	1.07±0.28	2.0×1010^−6^	2.2×10^−5^	0.89±0.30	0.74±0.33	0.763	0.842	0.86±0.40	0.71±0.23
EPA	0.15±0.13	0.22±0.09	0.002	0.010	0.14±0.24	0.12±0.13	0.180	0.262	0.10±0.10	0.06±0.05
DHA	0.35±0.28	0.59±0.27	0.002	0.010	0.30±0.35	0.31±0.29	0.157	0.251	0.22±0.24	0.17±0.13
Protein (E%)	18.0±3.1	18.5±2.3	0.210	0.280	19.1±3.2	18.8±2.5	0.950	0.980	18.8±3.7	18.3±2.5
Carbohydrate (E%)	46.6±7.1	48.1±5.8	0.194	0.270	45.6±6.3	47.2±7.5	0.268	0.343	47.8±5.6	47.3±5.1
Fiber (g)	29.2±8.2	36.4±6.3	4.3×10^−13^	1.4×10^−11^	24.6±7.0	26.5±5.4	2.7×10^−7^	4.3×10^−6^	22.5±7.0	18.0±4.2
Sucrose (E%)	7.2±2.7	6.2±2.0	0.121	0.204	5.8±2.6	5.3±2.6	0.619	0.707	6.2±2.7	5.9±2.2
Cholesterol (mg)	274±119	258±148	0.396	0.124	228±106	244±123	0.160	0.981	230±105	256±103
Alcohol (E%)	2.9±4.8	3.0±4.8	0.659	0.838	1.6±2.9	3.0±4.5	0.203	0.435	2.1±4.1	2.5±3.2

1 Based on a 4-day food record at baseline, ^2^Based on three 4-day food records (weeks 3, 7 and 11). ^3^Based on a mixed model comparison, where the interaction of time and group compared to Controls (time*group) is analyzed.

### Serum NMR metabolomics

**Figure 2:**
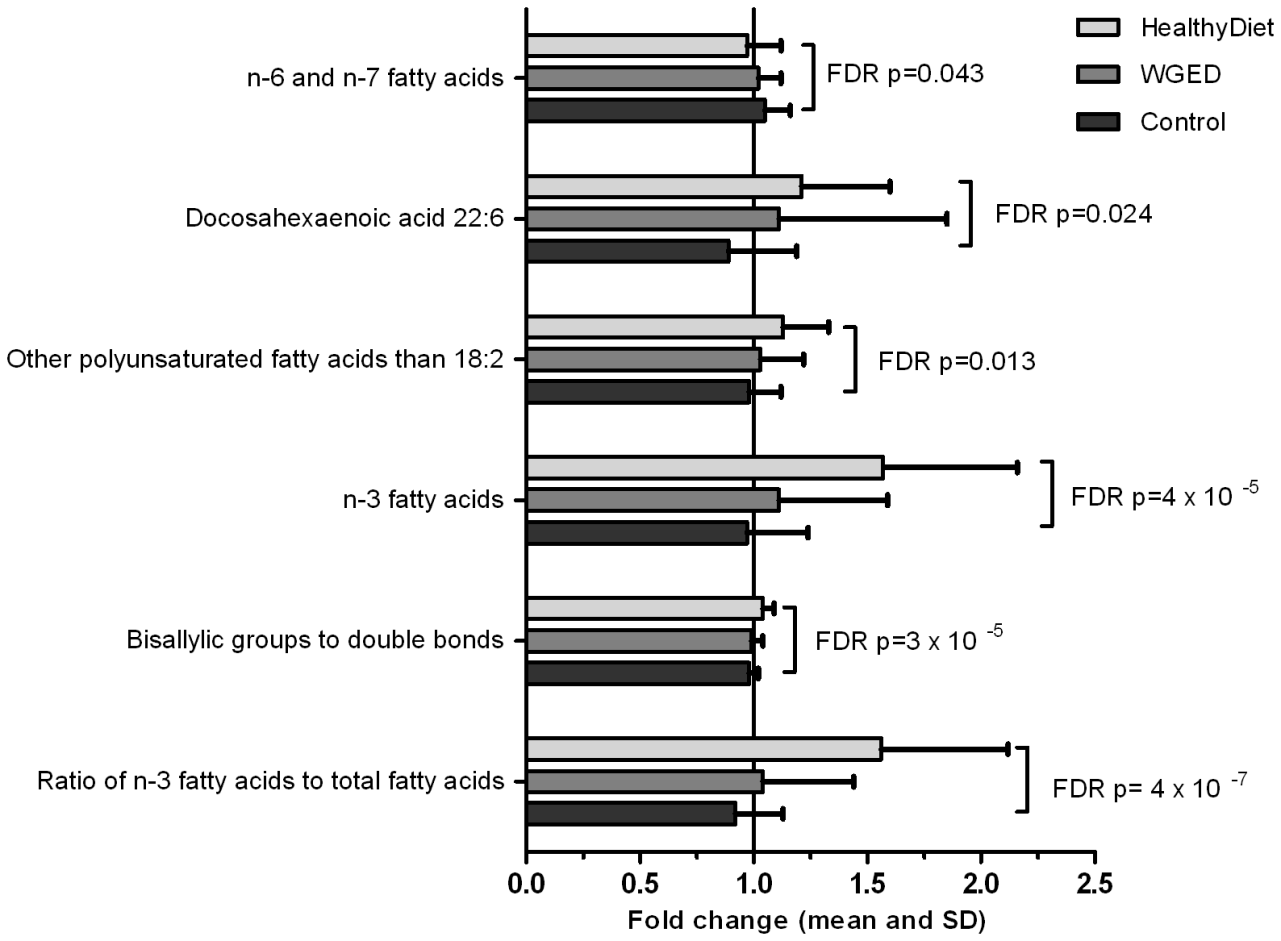
Fold changes in lipid variables at the end of the 12-week intervention period. Bars represent mean ± SD. FDR p-values are based on mixed model comparisons, where the interaction of time and group compared to Controls (time*group) or only the effect of time within a group is tested. HealthyDiet n =  37, Whole grain enriched diet (WGED) n =  34 and Control n = 32.

Mixed model analyses revealed significant differences in lipid metabolites when comparing HealthyDiet group to the Control group (Figure 2). All changes reflected increased polyunsaturation in plasma fatty acids. For example, ratio of bisallylic groups to double bonds (FDR p  =  3×10^−5^), ratio of bisallylic groups to total fatty acids (FDR p =  4×10^−4^) and average number of double bonds in fatty acid chain (FDR p =  0.024) increased and average number of methylene groups per double bonds decreased (FDR p =  0.012) in the HealthtyDiet group compared to the Control group. Bisallylic group has chemical structure of -CH = CH-CH2-CH = CH-, i.e. a single methylene group between the two double bonds and it reflects the level of polyunsaturation in fatty acids. Increases were seen especially in n-3 PUFAs, while n-6 and n-7 fatty acids decreased in the HealthyDiet group compared to the Control group, reflecting the good compliance to fish intake (Figure 2). The above mentioned differences existed also when comparing HealthyDiet group to WGED group. There were no significant changes in detected metabolites in the WGED compared to the Control group.

**Figure 3:**
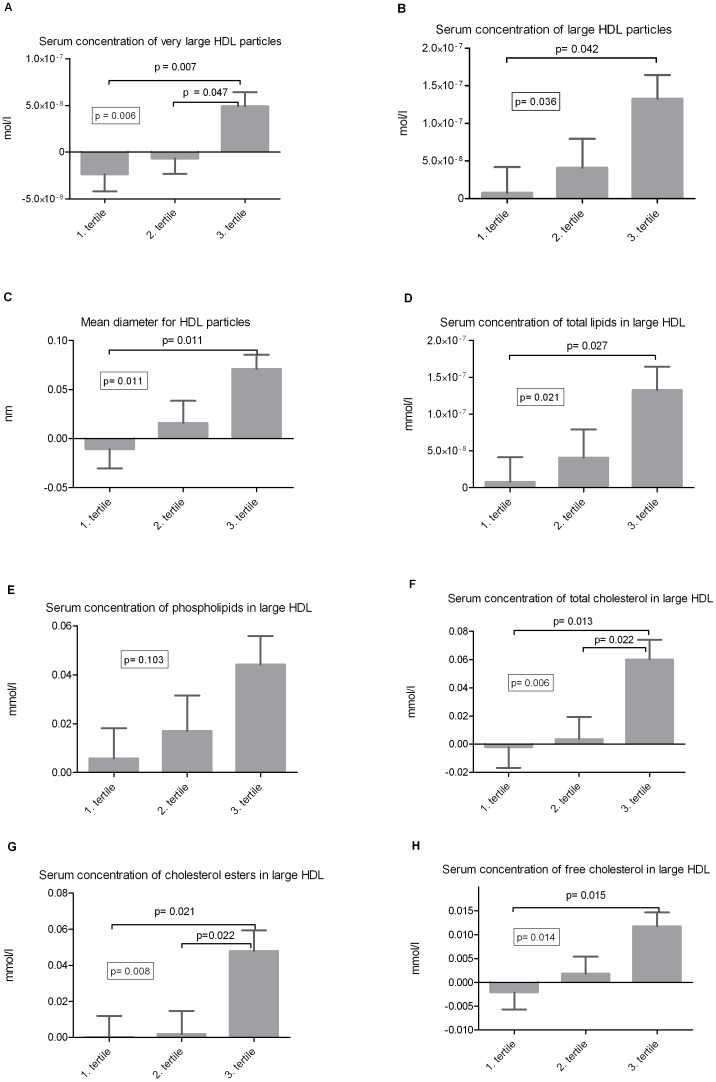
HDL particles and fatty fish intake. Changes in concentration of large HDL particles, mean particle diameter of HDL and serum concentrations of large HDL lipid components (mean ± SEM) according to tertiles of changes in fish intake. Changes in fish intake are calculated from 4-d food records which were kept once at baseline and three times during the intervention period.

There were no significant changes (FDR p < 0.05) in the metabolic measures quantified from the LIPO- and LMWM-windows (metabolites listed in the Supplemental table 1). However, in within-the-group comparisons we found and increasing trend (FDR p = 0.03 – 0.08) in variables related to large HDL particles in the HealthyDiet group (data not shown). The changes in concentrations of large HDL particles, in average diameter of HDL particles and in concentrations of lipid components of the large HDL particles associated positively with the changes in fish intake and changes in serum n-3 fatty acids concentrations (Table 3). When participants were divided into tertiles according to changes in fish intake we found that concentration of large HDL particles, mean diameter of HDL particles and serum concentrations of the lipid components of large HDL particles differed between these tertiles (Figure 3). The daily intake of fish decreased in the first tertile (average change: – 40 g/d, average intake during the intervention period: 26 g/d), remained nearly unchanged in the second tertile (average change: + 5 g/d, average intake: 28 g/d), and increased in the third tertile (average change: + 62 g/d, average intake: 74 g/d). The most of the consumed fish was fatty. Separation into tertiles based on changes in fish intake did not correspond exactly to the separation of intervention groups. However, most of the subjects (19 of 36) in the third tertile were from the HealthyDiet group as could be expected. There were no differences in changes in concentrations of total, LDL and HDL cholesterol and Tg between the tertiles (Figure S1).

**Table 3:** Associations^1^ between changes in fish intake, and changes in plasma n-3 fatty acid and DHA concentrations and changes in concentrations of very large and large HDL particles, average diameter of HDL particles and concentrations of large HDL lipid components in all study participants (n = 105).

	Total fish ^2^			Fatty fish ^2^			n-3 fatty acids ^3^			DHA ^3^		
	B	p	FDR p	B	p	FDR p	B	p	FDR p	B	p	FDR p
Concentration of very large HDL particles ^3^	0.269	0.011	0.019	0.232	0.030	0.117	0.414	0.001	0.008	0.277	0.020	0.038
Concentration of large HDL particles ^3^	0.268	0.012	0.019	0.178	0.102	0.117	0.318	0.011	0.016	0.271	0.024	0.038
Mean diameter for HDL particles ^3^	0.279	0.008	0.019	0.227	0.018	0.117	0.308	0.012	0.016	0.245	0.039	0.052
Total lipids in large HDL ^3^	0.268	0.012	0.019	0.178	0.102	0.117	0.318	0.011	0.016	0.271	0.024	0.038
Phospholipids in large HDL ^3^	0.258	0.017	0.023	0.154	0.164	0.164	0.248	0.050	0.050	0.202	0.098	0.098
Total cholesterol in large HDL ^3^	0.228	0.029	0.033	0.197	0.064	0.117	0.349	0.004	0.011	0.319	0.006	0.024
Cholesterol esters in large HDL ^3^	0.198	0.057	0.057	0.187	0.077	0.117	0.345	0.004	0.011	0.335	0.004	0.024
Free cholesterol in large HDL ^3^	0.288	0.007	0.019	0.199	0.068	0.117	0.307	0.014	0.016	0.221	0.068	0.078

1 Analysis of covariance (ANCOVA) using standardized variables. Age, BMI, gender, fasting glucose and group are included into the model, ^2^ Absolute change during the intervention, (g/d), ^3^ Changes in plasma concentration,

We also tested the cross-sectional associations at baseline and at the end of the study between mean diameters of HDL and LDL particles and the serum Tg concentration in all participants, and we found negative associations between the mean diameter of HDL and serum Tg concentration (beta = –0.410, p = 1.8×10^−5^ at baseline, beta = –0.320, p = 0.004 at the end), and the mean diameter of LDL and serum Tg concentration (beta = –0.380, p = 5.2×10^−6^ at baseline, beta = –0.572, p = 3.8×10^−10^ at the end). The associations were adjusted with BMI, age, gender and fasting glucose concentration at baseline and BMI, age, gender, fasting glucose concentration and intervention group at the end of the study.

### PON1 and lipid transfer protein activities

There were no significant changes in PLTP, CETP and PON1 activities during the intervention (Table 1). There was a slight positive association between CETP and PLTP activities and the variables related to extra-large HDL particles at baseline, but adjusted FDR p-values were not significant (Table 4). These associations were not seen at the end of the study. Further, we observed no correlations between PON1 activity and HDL particle variables at baseline or at the end of the study or between changes in lipid transfer protein activities and changes in HDL particle variables during the intervention period.

**Table 4:** Regressions^1^ between CETP, PLTP activities and variables related to HDL particles at baseline.

	CETP			PLTP			
	beta	p-value	FDR p	beta	p-value	FDR p	n
Fasting plasma HDL cholesterol (total)	0.051	0.640	0.668	0.144	0.190	0.304	106
Mean diameter for HDL particles	0.203	0.068	0.264	0.226	0.045	0.154	103
Concentration of very large HDL particles	0.220	0.042	0.240	0.227	0.037	0.148	103
Total lipids in very large HDL	0.213	0.050	0.240	0.243	0.026	0.148	101
Total cholesterol in very large HDL	0.230	0.031	0.240	0.273	0.011	0.132	101
Cholesterol esters in very large HDL	0.234	0.029	0.240	0.297	0.006	0.132	101
Free cholesterol in very large HDL	0.238	0.026	0.240	0.230	0.033	0.148	101
Phospholipids in very large HDL	0.193	0.077	0.264	0.201	0.068	0.204	101
Triglycerides in very large HDL	–0.001	0.991	0.991	0.238	0.030	0.148	103
Concentration of large HDL particles	0.102	0.363	0.484	0.163	0.150	0.285	103
Total lipids in large HDL	0.108	0.336	0.484	0.165	0.144	0.285	103
Total cholesterol in large HDL	0.119	0.282	0.484	0.171	0.125	0.285	103
Cholesterol esters in large HDL	0.107	0.334	0.484	0.175	0.115	0.285	103
Free cholesterol in large HDL	0.147	0.179	0.484	0.154	0.166	0.285	103
Phospholipids in large HDL	0.097	0.388	0.490	0.158	0.163	0.285	103
Concentration of medium HDL particles	–0.103	0.344	0.484	–0.001	0.990	0.990	103
Total lipids in medium HDL	–0.108	0.326	0.484	–0.012	0.912	0.990	101
Total cholesterol in medium HDL	–0.121	0.259	0.484	–0.037	0.737	0.931	101
Cholesterol esters in medium HDL	–0.127	0.233	0.484	–0.050	0.640	0.853	101
Free cholesterol in medium HDL	–0.080	0.471	0.514	0.005	0.968	0.990	101
Phospholipids in medium HDL	–0.081	0.467	0.514	0.020	0.856	0.978	101
Concentration of small HDL particles	–0.108	0.280	0.484	–0.100	0.323	0.485	103
Total lipids in small HDL	–0.103	0.305	0.484	–0.086	0.397	0.560	101
Triglycerides in small HDL	–0.070	0.471	0.514	0.023	0.814	0.977	101

1 Linear regression model; age, BMI, fasting glucose and gender included into the model.

## Discussion

In the present study the effects of diets containing fatty fish, wholegrain and low insulin response grain products, and bilberries on clinical characteristics, serum metabolites and lipid transfer protein activities in persons with impaired glucose metabolism were investigated. We found significant changes in lipid metabolites in the HealthyDiet group reflecting increased polyunsaturation of plasma fatty acids, particularly increase in n-3 PUFAs. Furthermore, in the within-the-group comparisons we found an increasing trend in variables related to large HDL particles in the HealthyDiet group, and when analysing this in more detail we found that the increase of fish intake correlated strongly with the increased concentration of large HDL particles, larger average diameter of HDL particles and increased concentrations of large HDL lipid components.

Significant changes in lipid metabolites in the HealthyDiet group were observed. All changes reflected increased polyunsaturation in plasma fatty acids, especially increase in n-3 PUFAs, while n-6 and n-7 fatty acids decreased. This is consistent with our earlier findings of increased plasma content of long chain polyunsaturated n-3 fatty acids and increase of plasma Tg incorporating the long chain PUFA in the HealthyDiet group [34]. These findings also confirm that different analytical methods (gas chromatography, UPLC-MS and ^1^H-NMR), give consistent results for comparable lipid measures. The observed changes confirm the good compliance to the instructions of fish intake, since the subjects in the HealthyDiet group were advised to eat fatty fish at least three times per week, whereas subjects in the Control group where instructed to restrict their fish intake for maximum of 1 fish meal per week. In the WGED group, dietary habits, besides the cereal products, were advised to keep unchanged, which did not cause changes in the measured metabolites.

Serum total, LDL and HDL cholesterol levels, serum Tg or serum apoA-1 and apoB100 concentrations did not change during the intervention. We observed that a greater increase of fish intake was associated with increased concentration of large HDL particles, larger average diameter of HDL particles and increased concentrations of lipid components in large HDL particles. This is consistent with earlier findings of increases in large HDL particles and/or decreases of small particles after consumptions of fatty fish or fish oil supplements [31]. The effects of n-3 PUFA on HDL metabolism has been studied in several randomized controlled trials, and the most of the studies suggest putative antiatherogenic changes, with increases of large HDL particles and/or decreases of small HDL particles [21], [31]. Nearly all studies have been conducted with fish oil supplements instead of fish consumption as part of the diet. It has also been shown that EPA and DHA supplementation improves reverse cholesterol transport (RCT) from macrophage to feces in hamsters fed with high fat diet [47]. Our findings of larger average diameter of HDL particles and increased concentration of large and extra-large HDL particles within the tertile with greatest increase in fish intake are consistent with earlier results [31]. We also observed an association between changes in fish intake and changes in concentrations of total lipids, phospholipids, total cholesterol, cholesterol esters and free cholesterol in large HDL. However, based on this data we are not able to conclude if this reflects changes in the content of large HDL particles or only increased serum concentration of large HDL particles. ApoA-1 and total HDL concentrations did not change during the intervention, which may be related to the trend of a decrease in small HDL particles (data not shown), that may compensate increases in larger particles.

Modern genome-wide and Mendelian randomization studies have failed to show a causal link between total HDL cholesterol concentration and coronary artery disease [48], [49], which might be related to the fact, that HDL is a heterogenous pool of subpopulations which differ in their structure and function [50]. In atherosclerosis-related diseases, variations in subpopulation levels and functions are observed, supporting the general view that the concentration of large HDL particles is inversely associated while that of small HDL particles is positively associated with cardiovascular disease [21], [31]. In addition, Gordon et al recently reported that the phospholipid content of large HDL subfractions showed a significant inverse correlation with the vascular stiffness assessed using pulse wave velocity [51]. In addition to this arterial stiffness measurements, cholesterol efflux from human THP-1 macrophages has been shown to correlate with phospholipids and particle mass of HDL demonstrating that the phospholipid content of HDL is an important determinant of cholesterol efflux [52]–[54]. Furthermore, the effect of HDL particle size on efflux capacity, and the impact of large HDL particles on cholesterol removal strongly suggest their protective role against the development of atherosclerosis [55], [56]. The association between hypertriglyceridemia and CVD risk is also controversial and is weakened when adjustment is made for other risk factors [48], [57]. Here we found cross-sectional negative correlations between serum Tg concentration and average diameter of HDL and LDL particles, a finding that is also evident in metabolic syndrome [58]. This suggests that the known effect of serum Tg concentration on increased risk of CVD or type 2 diabetes may in part be related to altered HDL metabolism [35].

The relationship between CETP and lipoprotein metabolism is complex [59], [60]. CETP is central in cholesterol and Tg transport between HDL and apoB-containing lipoproteins in the circulation, and this CETP-mediated lipid transfer clearly differs in various dyslipidemias [61]. CETP simultaneously affects the concentration and composition of both antiatherogenic and atherogenic lipoproteins and its connection to coronary heart disease risk is not completely understood [61]. No changes in the lipid transfer protein activities during the 12-week dietary intervention were observed. Earlier results of the effects of fish oil treatment on CETP activity are discordant, which might be related to the type of fish oil studied (EPA vs. DHA) [62], [63]. We found weak positive association between CETP and PLTP activities and mean particle diameter of HDL and other variables related to extra-large HDL particles at baseline. Overall, CETP can be seen as one among the factors affecting reverse cholesterol transport pathway and high CETP activity is reportedly associated with decreased HDL cholesterol levels [61]. CETP inhibitors have recently gained great interest due to their ability to increase HDL cholesterol concentration, but it remains to be seen whether their use will result in functionally protective HDL populations and clinical benefit in large trials [64].

Although the effects of intervention on clinical characteristics and measured metabolites were modest, we found a strong association between increased fish intake and variables related to large HDL particles. However, as a potential limitation, our study design does not allow for total separation of fish intake from increased intake of whole grain and low insulin response grain products and bilberries, but this was considered in statistical analyses by including the intervention group into the model. In fact, not all subjects in the third tertile with the greatest increase in fish intake were in the HealthyDiet group. The average consumption of fish was 74 grams per day in the third tertile, which is more than 500 g per week. This suggests that the instructed three fish meals per week were not enough for these changes. The mean consumption of fish in the third tertile was more than double of average fish consumption among the Finnish population [65]. We have seen that people who are willing to participate in dietary interventions are more interested in healthy eating than people in average. This phenomenon is also observed when comparing the baseline nutrient intakes in the present study with the respective average intakes in Finland (e.g. lower intake of SFA and sucrose and higher intake of dietary fiber in the study population) [65]. This is one obvious explanation why it might be difficult to see significant changes in the intervention groups with healthy diet. For the same reason, people in the Control group had to limit their rye bread, fish and berry intake, so that the control diet resembled more an average Finnish diet. For example, rye bread intake in men in the Northern Savo area is more than double compared to corresponding intake in Southern Finland [65].

Besides lipoprotein subclass distribution and detailed information on serum lipids, the NMR platform enables detection of low-molecular-weight metabolites including e.g. citrate, acetate, urea and amino acids. We did not see changes in these 22 measured metabolites during the intervention. The battery of 22 low-molecular-weight metabolites was not selected particularly for this dietary intervention study and we did not have preliminary hypothesis related to these metabolites. Furthermore, the power calculation was based on fasting glucose concentration, and it is possible that there was not enough power to see all changes in these secondary outcome variables. Of these metabolites, branched chain amino acids were probably the most interesting ones, because they are related to type 2 diabetes risk and we have earlier found that serum isoleucine decreased after the 12 week consumption of diet high in rye bread and pasta [66], [67]. Despite similar carbohydrate modification of the diet in the HealthyDiet and WGED groups of the present study (increasing the intakes of rye bread and pasta), we could not see any changes in isoleucine. This confirms our earlier conclusion that decreased isoleucine may rather be due to decreased intake of protein rich foods, which are also sources of isoleucine, than the intervention itself. In the current study, there were no changes in protein intake during the intervention period.

In conclusion, increased fatty fish intake was associated with beneficially considered changes in HDL particles in persons at risk for coronary heart disease. The changes which we saw in large HDL particles could be related to those parameters that are functionally related to reverse cholesterol transport. Thus, our findings could partly explain the known protective effects of fish consumption against the atherosclerosis.

## Supporting Information

Figure S1 (TIF)

Table S1 (DOCX)

Checklist S1 (DOC)

Finnish publication S1 (PDF)

Method S1 (DOCX)

Protocol S1 (DOCX)

## References

[1] Ye EQ, Chacko SA, Chou EL, Kugizaki M, Liu S (2012) Greater whole-grain intake is associated with lower risk of type 2 diabetes, cardiovascular disease, and weight gain. J Nutr 142: 1304–1313. 22649266 10.3945/jn.111.155325 PMC6498460

[2] Musa-Veloso K, Binns MA, Kocenas A, Chung C, Rice H, et al (2011) Impact of low v. moderate intakes of long-chain n-3 fatty acids on risk of coronary heart disease. Br J Nutr 106: 1129–1141. 21736820 10.1017/S0007114511001644

[3] Basu A, Rhone M, Lyons TJ (2010) Berries: emerging impact on cardiovascular health. Nutr Rev 68: 168–177. 20384847 10.1111/j.1753-4887.2010.00273.x PMC3068482

[4] Olsen A, Egeberg R, Halkjaer J, Christensen J, Overvad K, Tjonneland A (2011) Healthy aspects of the Nordic diet are related to lower total mortality. J Nutr 141: 639–644. 21346102 10.3945/jn.110.131375

[5] Adamsson V, Reumark A, Fredriksson IB, Hammarstrom E, Vessby B, et al (2011) Effects of a healthy Nordic diet on cardiovascular risk factors in hypercholesterolaemic subjects: a randomized controlled trial (NORDIET). J Intern Med 269: 150–159. 20964740 10.1111/j.1365-2796.2010.02290.x

[6] de Mello VD, Schwab U, Kolehmainen M, Koenig W, Siloaho M, et al (2011) A diet high in fatty fish, bilberries and wholegrain products improves markers of endothelial function and inflammation in individuals with impaired glucose metabolism in a randomised controlled trial: the Sysdimet study. Diabetologia 54: 2755–2767. 21870174 10.1007/s00125-011-2285-3

[7] Uusitupa M, Hermansen K, Savolainen MJ, Schwab U, Kolehmainen M, et al (2013) Effects of an isocaloric healthy Nordic diet on insulin sensitivity, lipid profile and inflammation markers in metabolic syndrome - a randomized study (SYSDIET). J Intern Med 274: 52–66. 23398528 10.1111/joim.12044 PMC3749468

[8] Sofi F, Cesari F, Abbate R, Gensini GF, Casini A (2008) Adherence to Mediterranean diet and health status: meta-analysis. BMJ 337: a1344. 18786971 10.1136/bmj.a1344 PMC2533524

[9] Fung TT, Rexrode KM, Mantzoros CS, Manson JE, Willett WC, Hu FB (2009) Mediterranean diet and incidence of and mortality from coronary heart disease and stroke in women. Circulation 119: 1093–1100. 19221219 10.1161/CIRCULATIONAHA.108.816736 PMC2724471

[10] Salas-Salvado J, Bullo M, Babio N, Martinez-Gonzalez MA, Ibarrola-Jurado N, et al (2011) Reduction in the incidence of type 2 diabetes with the Mediterranean diet: results of the PREDIMED-Reus nutrition intervention randomized trial. Diabetes Care 34: 14–19. 20929998 10.2337/dc10-1288 PMC3005482

[11] He K, Song Y, Daviglus ML, Liu K, Van Horn L, et al (2004) Accumulated evidence on fish consumption and coronary heart disease mortality: a meta-analysis of cohort studies. Circulation 109: 2705–2711. 15184295 10.1161/01.CIR.0000132503.19410.6B

[12] Harris WS, Kris-Etherton PM, Harris KA (2008) Intakes of long-chain omega-3 fatty acid associated with reduced risk for death from coronary heart disease in healthy adults. Curr Atheroscler Rep 10: 503–509. 18937898 10.1007/s11883-008-0078-z

[13] Harris WS (1997) N-3 Fatty Acids and Serum Lipoproteins: Human Studies. Am J Clin Nutr 65: 1645S–1654S. 9129504 10.1093/ajcn/65.5.1645S

[14] Balk EM, Lichtenstein AH, Chung M, Kupelnick B, Chew P, Lau J (2006) Effects of omega-3 fatty acids on serum markers of cardiovascular disease risk: a systematic review. Atherosclerosis 189: 19–30. 16530201 10.1016/j.atherosclerosis.2006.02.012

[15] Kwak SM, Myung SK, Lee YJ, Seo HG (2012) Korean Meta-analysis Study Group (2012) Efficacy of omega-3 fatty acid supplements (eicosapentaenoic acid and docosahexaenoic acid) in the secondary prevention of cardiovascular disease: a meta-analysis of randomized, double-blind, placebo-controlled trials. Arch Intern Med 172: 686–694. 22493407 10.1001/archinternmed.2012.262

[16] Karlstrom BE, Jarvi AE, Byberg L, Berglund LG, Vessby BO (2011) Fatty fish in the diet of patients with type 2 diabetes: comparison of the metabolic effects of foods rich in n-3 and n-6 fatty acids. Am J Clin Nutr 94: 26–33. 21613555 10.3945/ajcn.110.006221

[17] Akinkuolie AO, Ngwa JS, Meigs JB, Djousse L (2011) Omega-3 polyunsaturated fatty acid and insulin sensitivity: a meta-analysis of randomized controlled trials. Clin Nutr 30: 702–707. 21959352 10.1016/j.clnu.2011.08.013 PMC5066815

[18] Brennan L (2013) Metabolomics in nutrition research: current status and perspectives. Biochem Soc Trans 41: 670–673. 23514174 10.1042/BST20120350

[19] Soininen P, Kangas AJ, Wurtz P, Tukiainen T, Tynkkynen T, et al (2009) High-throughput serum NMR metabonomics for cost-effective holistic studies on systemic metabolism. Analyst 134: 1781–1785. 19684899 10.1039/b910205a

[20] Kettunen J, Tukiainen T, Sarin AP, Ortega-Alonso A, Tikkanen E, et al (2012) Genome-wide association study identifies multiple loci influencing human serum metabolite levels. Nat Genet 44: 269–276. 22286219 10.1038/ng.1073 PMC3605033

[21] Pirillo A, Norata GD, Catapano AL (2013) High-density lipoprotein subfractions—what the clinicians need to know. Cardiology 124: 116–125. 23428644 10.1159/000346463

[22] Arsenault BJ, Lemieux I, Despres JP, Gagnon P, Wareham NJ, et al (2009) HDL particle size and the risk of coronary heart disease in apparently healthy men and women: the EPIC-Norfolk prospective population study. Atherosclerosis 206: 276–281. 19268944 10.1016/j.atherosclerosis.2009.01.044

[23] Villard EF, El Khoury P, Duchene E, Bonnefont-Rousselot D, Clement K, et al (2012) Elevated CETP activity improves plasma cholesterol efflux capacity from human macrophages in women. Arterioscler Thromb Vasc Biol 32: 2341–2349. 22904275 10.1161/ATVBAHA.112.252841

[24] Villard EF, EI Khoury P, Frisdal E, Bruckert E, Clement K, et al (2013) Genetic determination of plasma cholesterol efflux capacity is gender-specific and independent of HDL-cholesterol levels. Arterioscler Thromb Vasc Biol 33: 822–828. 23372063 10.1161/ATVBAHA.112.300979

[25] Vikstedt R, Metso J, Hakala J, Olkkonen VM, Ehnholm C, Jauhiainen M (2007) Cholesterol efflux from macrophage foam cells is enhanced by active phospholipid transfer protein through generation of two types of acceptor particles. Biochemistry 46: 11979–11986. 17900150 10.1021/bi700833h

[26] Aviram M, Vaya J (2013) Paraoxonase 1 activities, regulation, and interactions with atherosclerotic lesion. Curr Opin Lipidol 10.1097/MOL.0b013e32835ffcfd 23508039

[27] Mukamal KJ, Mackey RH, Kuller LH, Tracy RP, Kronmal RA, et al (2007) Alcohol consumption and lipoprotein subclasses in older adults. J Clin Endocrinol Metab 92: 2559–2566. 17440017 10.1210/jc.2006-2422

[28] Sonestedt E, Wirfalt E, Wallstrom P, Gullberg B, Drake I, et al (2012) High disaccharide intake associates with atherogenic lipoprotein profile. Br J Nutr 107: 1062–1069. 22011476 10.1017/S0007114511003783

[29] Bogl LH, Maranghi M, Rissanen A, Kaprio J, Taskinen MR, Pietilainen KH (2011) Dietary omega-3 polyunsaturated fatty acid intake is related to a protective high-density lipoprotein subspecies profile independent of genetic effects: a monozygotic twin pair study. Atherosclerosis 219: 880–886. 21962401 10.1016/j.atherosclerosis.2011.09.010

[30] Annuzzi G, Rivellese AA, Wang H, Patti L, Vaccaro O, et al (2012) Lipoprotein subfractions and dietary intake of n-3 fatty acid: the Genetics of Coronary Artery Disease in Alaska Natives study. Am J Clin Nutr 95: 1315–1322. 22572646 10.3945/ajcn.111.023887 PMC3349453

[31] Burillo E, Martin-Fuentes P, Mateo-Gallego R, Baila-Rueda L, Cenarro A, et al (2012) Omega-3 fatty acids and HDL. How do they work in the prevention of cardiovascular disease? Curr Vasc Pharmacol 10: 432–441. 22339302 10.2174/157016112800812845

[32] Erkkilä A, Schwab U, Lehto S, de Mello V, Kangas A, et al. (2013, Article in press.) Effect of fatty and lean fish intake on lipoprotein subclasses in subjects with coronary heart disease: A controlled trial. Journal of Clinical lipidology. 10.1016/j.jacl.2013.09.007 24528693

[33] Bogl LH, Pietilainen KH, Rissanen A, Kangas AJ, Soininen P, et al (2013) Association between habitual dietary intake and lipoprotein subclass profile in healthy young adults. Nutr Metab Cardiovasc Dis 23: 1071–8. 23333726 10.1016/j.numecd.2012.11.007

[34] Lankinen M, Schwab U, Kolehmainen M, Paananen J, Poutanen K, et al (2011) Whole Grain Products, Fish and Bilberries Alter Glucose and Lipid Metabolism in a Randomized, Controlled Trial: The Sysdimet Study. PLoS ONE 6: e22646. 21901116 10.1371/journal.pone.0022646 PMC3161986

[35] Wang J, Stancakova A, Soininen P, Kangas AJ, Paananen J, et al (2012) Lipoprotein subclass profiles in individuals with varying degrees of glucose tolerance: a population-based study of 9399 Finnish men. J Intern Med 272: 562–572. 22650159 10.1111/j.1365-2796.2012.02562.x

[36] Inouye M, Kettunen J, Soininen P, Silander K, Ripatti S, et al (2010) Metabonomic, transcriptomic, and genomic variation of a population cohort. Mol Syst Biol 6: 441. 21179014 10.1038/msb.2010.93 PMC3018170

[37] Wurtz P, Makinen VP, Soininen P, Kangas AJ, Tukiainen T, et al (2012) Metabolic signatures of insulin resistance in 7,098 young adults. Diabetes 61: 1372–1380. 22511205 10.2337/db11-1355 PMC3357275

[38] Kujala UM, Makinen VP, Heinonen I, Soininen P, Kangas AJ, et al (2013) Long-term leisure-time physical activity and serum metabolome. Circulation 127: 340–348. 23258601 10.1161/CIRCULATIONAHA.112.105551

[39] Tukiainen T, Kettunen J, Kangas AJ, Lyytikainen LP, Soininen P, et al (2012) Detailed metabolic and genetic characterization reveals new associations for 30 known lipid loci. Hum Mol Genet 21: 1444–1455. 22156771 10.1093/hmg/ddr581

[40] Chambers JC, Zhang W, Sehmi J, Li X, Wass MN, et al (2011) Genome-wide association study identifies loci influencing concentrations of liver enzymes in plasma. Nat Genet 43: 1131–1138. 22001757 10.1038/ng.970 PMC3482372

[41] Groener JE, Pelton RW, Kostner GM (1986) Improved estimation of cholesteryl ester transfer/exchange activity in serum or plasma. Clin Chem 32: 283–286. 3943188

[42] Jauhiainen M, Ehnholm C (2005) Determination of human plasma phospholipid transfer protein mass and activity. Methods 36: 97–101. 15893933 10.1016/j.ymeth.2004.11.006

[43] Kleemola P, Freese R, Jauhiainen M, Pahlman R, Alfthan G, Mutanen M (2002) Dietary determinants of serum paraoxonase activity in healthy humans. Atherosclerosis 160: 425–432. 11849667 10.1016/s0021-9150(01)00594-9

[44] R Development Core Team R: A language and environment for statistical computing. [Homepage of R Foundation for Statistical Computing,Vienna, Austria], 2010, Available: http://www.R-project.org.

[45] Pinheiro J, Bates D, DebRoy S, Sarkar D & the R Core team. nlme: Linear and Nonlinear Mixed Effects Models. 2009.

[46] Benjamini Y, Hochberg Y (1995) Controlling the false discovery rate: a practical and powerful approach to multiple testing. J R Statist Soc B 57: 289–300.

[47] Kasbi Chadli F, Nazih H, Krempf M, Nguyen P, Ouguerram K (2013) Omega 3 Fatty acids promote macrophage reverse cholesterol transport in hamster fed high fat diet. PLoS One 8: e61109. 23613796 10.1371/journal.pone.0061109 PMC3632549

[48] CARDIoGRAMplusC4D Consortium, Deloukas P, Kanoni S, Willenborg C, Farrall M, et al (2013) Large-scale association analysis identifies new risk loci for coronary artery disease. Nat Genet 45: 25–33. 23202125 10.1038/ng.2480 PMC3679547

[49] Haase CL, Tybjaerg-Hansen A, Qayyum AA, Schou J, Nordestgaard BG, Frikke-Schmidt R (2012) LCAT, HDL cholesterol and ischemic cardiovascular disease: a Mendelian randomization study of HDL cholesterol in 54,500 individuals. J Clin Endocrinol Metab 97: E248–56. 22090275 10.1210/jc.2011-1846

[50] Rye KA, Bursill CA, Lambert G, Tabet F, Barter PJ (2009) The metabolism and anti-atherogenic properties of HDL. J Lipid Res 50 Suppl:S195–200 10.1194/jlr.R800034-JLR200 PMC2674714 19033213

[51] Gordon SM, Davidson WS, Urbina EM, Dolan LM, Heink A, et al (2013) The effects of type 2 diabetes on lipoprotein composition and arterial stiffness in male youth. Diabetes 62: 2958–2967. 23835332 10.2337/db12-1753 PMC3717874

[52] Nakanishi S, Vikstedt R, Soderlund S, Lee-Rueckert M, Hiukka A, et al (2009) Serum, but not monocyte macrophage foam cells derived from low HDL-C subjects, displays reduced cholesterol efflux capacity. J Lipid Res 50: 183–192. 18787236 10.1194/jlr.M800196-JLR200

[53] Gelissen IC, Harris M, Rye KA, Quinn C, Brown AJ, et al (2006) ABCA1 and ABCG1 synergize to mediate cholesterol export to apoA-I. Arterioscler Thromb Vasc Biol 26: 534–540. 16357317 10.1161/01.ATV.0000200082.58536.e1

[54] Fournier N, Paul JL, Atger V, Cogny A, Soni T, et al (1997) HDL phospholipid content and composition as a major factor determining cholesterol efflux capacity from Fu5AH cells to human serum. Arterioscler Thromb Vasc Biol 17: 2685–2691. 9409243 10.1161/01.atv.17.11.2685

[55] Davidson WS, Rodrigueza WV, Lund-Katz S, Johnson WJ, Rothblat GH, Phillips MC (1995) Effects of acceptor particle size on the efflux of cellular free cholesterol. J Biol Chem 270: 17106–17113. 7615505 10.1074/jbc.270.29.17106

[56] Matsuura F, Wang N, Chen W, Jiang XC, Tall AR (2006) HDL from CETP-deficient subjects shows enhanced ability to promote cholesterol efflux from macrophages in an apoE- and ABCG1-dependent pathway. J Clin Invest 116: 1435–1442. 16670775 10.1172/JCI27602 PMC1451209

[57] Graham I, Cooney MT, Bradley D, Dudina A, Reiner Z (2012) Dyslipidemias in the prevention of cardiovascular disease: risks and causality. Curr Cardiol Rep 14: 709–720. 22965836 10.1007/s11886-012-0313-7

[58] Soderlund S, Watanabe H, Ehnholm C, Jauhiainen M, Taskinen MR (2010) Increased apolipoprotein E level and reduced high-density lipoprotein mean particle size associate with low high-density lipoprotein cholesterol and features of metabolic syndrome. Metabolism 59: 1502–1509. 20206948 10.1016/j.metabol.2010.01.015

[59] Parra ES, Urban A, Panzoldo NB, Nakamura RT, Oliveira R, de Faria EC (2011) A reduction of CETP activity, not an increase, is associated with modestly impaired postprandial lipemia and increased HDL-cholesterol in adult asymptomatic women. Lipids Health Dis 10: 87–511X-10–87. 21609439 10.1186/1476-511X-10-87 PMC3125351

[60] Barter PJ, Rye KA (2012) Cholesteryl ester transfer protein inhibition as a strategy to reduce cardiovascular risk. J Lipid Res 53: 1755–1766. 22550134 10.1194/jlr.R024075 PMC3413218

[61] de Grooth GJ, Klerkx AH, Stroes ES, Stalenhoef AF, Kastelein JJ, Kuivenhoven JA (2004) A review of CETP and its relation to atherosclerosis. J Lipid Res 45: 1967–1974. 15342674 10.1194/jlr.R400007-JLR200

[62] Abbey M, Clifton P, Kestin M, Belling B, Nestel P (1990) Effect of fish oil on lipoproteins, lecithin:cholesterol acyltransferase, and lipid transfer protein activity in humans. Arteriosclerosis 10: 85–94. 2297349 10.1161/01.atv.10.1.85

[63] Homma Y, Ohshima K, Yamaguchi H, Nakamura H, Araki G, Goto Y (1991) Effects of eicosapentaenoic acid on plasma lipoprotein subfractions and activities of lecithin:cholesterol acyltransferase and lipid transfer protein. Atherosclerosis 91: 145–153. 1811549 10.1016/0021-9150(91)90196-a

[64] Bishop BM (2013) Systematic Review of CETP Inhibitors for Increasing High-Density Lipoprotein Cholesterol: Where Do These Agents Stand in the Approval Process? Am J Ther 10.1097/MJT.0b013e31828b8463 23567794

[65] Paturi M, Tapanainen H, Reinivuo H, Pietinen P (2008) The National FINDIET 2007 Survey. Publications of the National Public Health Institute B 23

[66] Wang TJ, Larson MG, Vasan RS, Cheng S, Rhee EP, et al (2011) Metabolite profiles and the risk of developing diabetes. Nat Med 17: 448–453. 21423183 10.1038/nm.2307 PMC3126616

[67] Lankinen M, Schwab U, Gopalacharyulu PV, Seppanen-Laakso T, Yetukuri L, et al (2010) Dietary carbohydrate modification alters serum metabolic profiles in individuals with the metabolic syndrome. Nutr Metab Cardiovasc Dis 20: 249–257. 19553094 10.1016/j.numecd.2009.04.009

