# Supplementary figures and images for: Effects of Whole Grain, Fish and Bilberries on Serum Metabolic Profile and Lipid Transfer Protein Activities: A Randomized Trial (Sysdimet)
Source: PLoS One. 2014 Feb 28;9(2):e90352. doi: 10.1371/journal.pone.0090352 (PMC3938672; doi:10.1371/journal.pone.0090352)

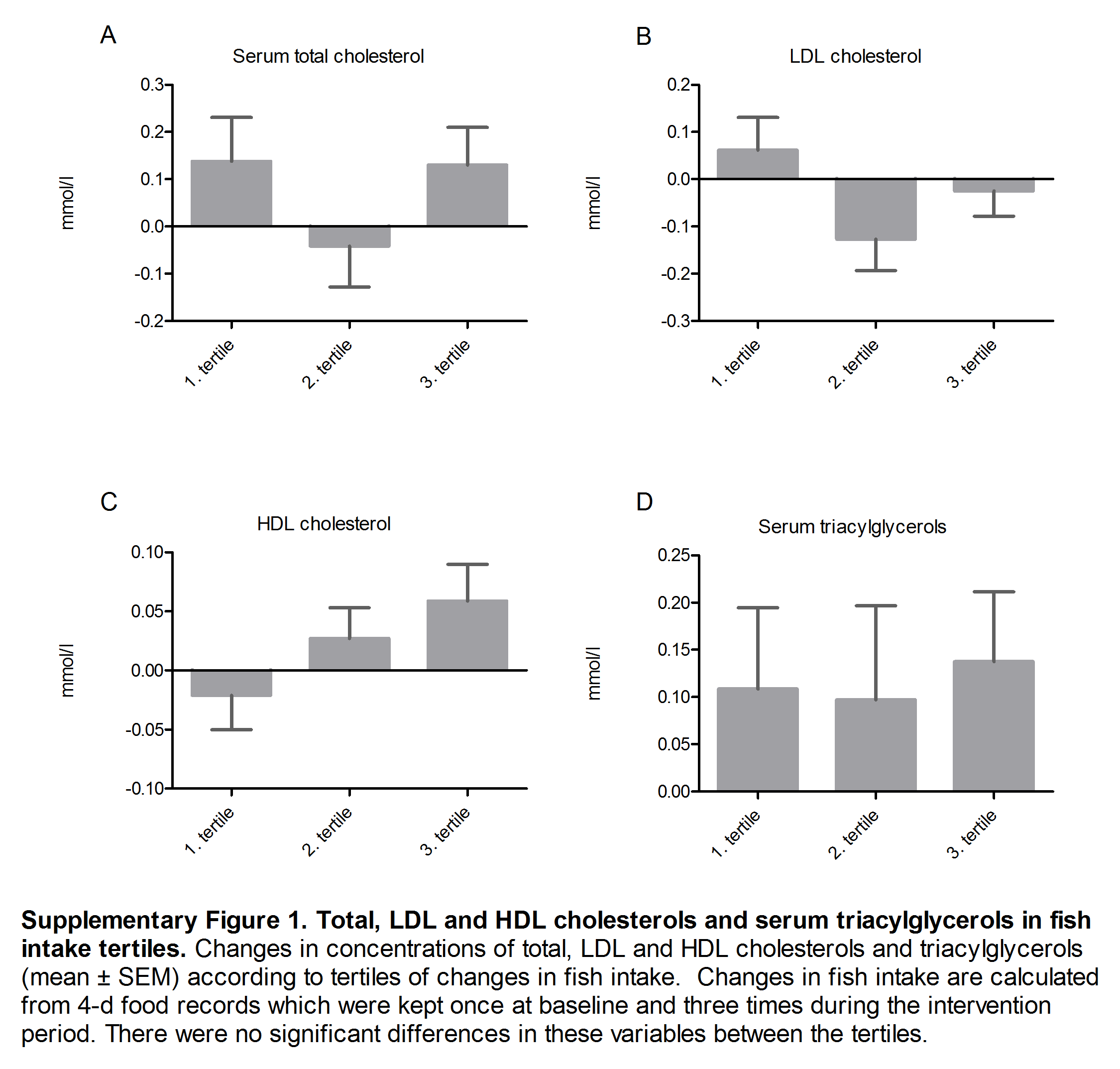

Supplement: Figure S1 — (TIF) [file pone.0090352.s001.tif]
