# Supplementary material for: Effects of Whole Grain, Fish and Bilberries on Serum Metabolic Profile and Lipid Transfer Protein Activities: A Randomized Trial (Sysdimet)
Source: PLoS One. 2014 Feb 28;9(2):e90352. doi: 10.1371/journal.pone.0090352 (PMC3938672; doi:10.1371/journal.pone.0090352)
Supplement: Table S1 — (DOCX) [file pone.0090352.s002.docx]

Supplementary Table S1. Serum NMR metabolite list.

| **LIPO window** | |
| --- | --- |
| Albumin | Concentration of medium LDL particles |
| Phospholipids in chylomicrons and extremely large VLDL | Total cholesterol in small LDL |
| Total lipids in chylomicrons and extremely large VLDL | Total lipids in small LDL |
| Concentration of chylomicrons and extremely large VLDL particles | Concentration of small LDL particles |
| Phospholipids in very large VLDL | Total cholesterol in very large HDL |
| Triglycerides in very large VLDL | Free cholesterol in very large HDL |
| Total lipids in very large VLDL | Phospholipids in very large HDL |
| Concentration of very large VLDL particles | Triglycerides in very large HDL |
| Total cholesterol in large VLDL | Cholesterol esters in very large HDL |
| Free cholesterol in large VLDL | Total lipids in very large HDL |
| Phospholipids in large VLDL | Concentration of very large HDL particles |
| Triglycerides in large VLDL | Total cholesterol in large HDL |
| Cholesterol esters in large VLDL | Free cholesterol in large HDL |
| Total lipids in large VLDL | Phospholipids in large HDL |
| Concentration of large VLDL particles | Cholesterol esters in large HDL |
| Total cholesterol in medium VLDL | Total lipids in large HDL |
| Free cholesterol in medium VLDL | Concentration of large HDL particles |
| Phospholipids in medium VLDL | Total cholesterol in medium HDL |
| Triglycerides in medium VLDL | Free cholesterol in medium HDL |
| Cholesterol esters in medium VLDL | Phospholipids in medium HDL |
| Total lipids in medium VLDL | Cholesterol esters in medium HDL |
| Concentration of medium VLDL particles | Total lipids in medium HDL |
| Total cholesterol in small VLDL | Concentration of medium HDL particles |
| Free cholesterol in small VLDL | Triglycerides in small HDL |
| Phospholipids in small VLDL | Total lipids in small HDL |
| Triglycerides in small VLDL | Concentration of small HDL particles |
| Total lipids in small VLDL | Triglycerides in chylomicrons and extremely large VLDL |
| Concentration of small VLDL particles | Triglycerides in VLDL |
| Phospholipids in very small VLDL | Triglycerides in IDL |
| Triglycerides in very small VLDL | Total cholesterol in IDL |
| Total lipids in very small VLDL | Total cholesterol in LDL |
| Concentration of very small VLDL particles | Total cholesterol in HDL |
| Free cholesterol in IDL | Serum total triglycerides |
| Phospholipids in IDL | Serum total cholesterol |
| Total lipids in IDL | Mean diameter for VLDL particles |
| Concentration of IDL particles | Mean diameter for LDL particles (includes IDL particles) |
| Total cholesterol in large LDL | Mean diameter for HDL particles |
| Free cholesterol in large LDL | Triglycerides in VLDL (Lipido) |
| Phospholipids in large LDL | Total cholesterol in IDL (Lipido) |
| Cholesterol esters in large LDL | Total cholesterol in LDL (Lipido) |
| Total lipids in large LDL | Total cholesterol in HDL3 (Lipido) |
| Concentration of large LDL particles | Apolipoprotein A-I (Lipido) |
| Total cholesterol in medium LDL | Apolipoprotein B (Lipido) |
| Phospholipids in medium LDL | Apolipoprotein B by apolipoprotein A-I (Lipido) |
| Cholesterol esters in medium LDL | Total cholesterol in HDL3 (Lipido) |
| Total lipids in medium LDL |  |
| **LIPID window** | |
| Total cholesterol | Sphingomyelins |
| Esterified cholesterol | Total cholines (and other N-trimethyl compounds) |
| Free cholesterol | Ratio of omega-3 fatty acids to total fatty acids |
| Omega-3 fatty acids | Ratio of omega-6/7 fatty acids to total fatty acids |
| Omega-6 and -7 fatty acids | Ratio of omega-9 and saturated fatty acids to total fatty acids |
| Omega-9 and saturated fatty acids | Average number of methylene groups in a fatty acid chain |
| Total fatty acids | Ratio of triglycerides to phosphoglycerides |
| 18:3. linoleic acid (LA) | Average number of methylene groups per a double bond |
| Other polyunsaturated fatty acids than 18:3 | Average number of double bonds in a fatty acid chain |
| 33:6. docosahexaenoic acid (DHA) | Ratio of bisallylic groups to double bonds |
| Total triglycerides | Ratio of bisallylic groups to total fatty acids |
| Total phosphoglycerides | Description of average fatty acid chain length (not actual carbon number) |
| Phosphatidylcholine (and other cholines) |  |
| **LMWM window** | |
| 3-hydroxybutyrate | Glycoproteins |
| Acetate | Histidine |
| Acetoacetate | Isoleucine |
| Alanine | Lactate |
| Mobile lipids -CH3- | Leucine |
| Mobile lipids -CH3 | Phenylalanine |
| Citrate | Pyruvate |
| Creatinine | Tyrosine |
| Glucose | Double bond protons of mobile lipids |
| Glutamine | Urea |
| Glycerol | Valine |
