# Supplementary material for: Effects of Whole Grain, Fish and Bilberries on Serum Metabolic Profile and Lipid Transfer Protein Activities: A Randomized Trial (Sysdimet)
Source: PLoS One. 2014 Feb 28;9(2):e90352. doi: 10.1371/journal.pone.0090352 (PMC3938672; doi:10.1371/journal.pone.0090352)
Supplement: Finnish publication S1 — (PDF) [file pone.0090352.s004.pdf]

Laura Joukamo, Maria Lankinen, Ursula Schwab, Pasi Soininen, Antti J. Kangas, Marjukka Kolehmainen, Jussi Paananen, Kaisa Poutanen, Hannu Mykkänen, Tuulikki Seppänen-Laakso, Helena Gylling, Matej Orešič, Mika Ala-Korpela ja Matti Uusitupa

Rasvainen kala muokkaa HDL-hiukkaskokoa ja lipidipitoisuuksia

TAUSTA: Osana laajempaa Sysdimet-ruokavaliotutkimusta selvitettiin ^{1}HNMR-spektroskopian avulla rasvaisen kalan vaikutuksia seerumin HDL-lipoproteiinipartikkelialaluokkiin henkilöillä, joilla oli riskitekijäkasauma.

AINEISTO JA MENETELMÄT: Tutkittavat (n = 105) satunnaistettiin kolmeen ryhmään. Ensimmäisessä lisättiin rasvaisen kalan, täysjyväviljan ja mustikoiden kulutusta (kaladieetti, n = 37). Toisessa kalan kulutus pidettiin ennallaan, mutta lisättiin täysjyväviljan määrää (täysjyvä, n = 34). Kolmas ryhmä puolestaan rajoitti kalan kulutustaan (verrokkiryhmä, n = 35). Tutkittavat pitivät toistuvasti ruokapäiväkirjaa, ja heistä otettiin seeruminäytteet ennen ja jälkeen 12-viikkoisen tutkimuksen. Lipoproteiinipartikkelit analysoitiin ^{1}HNMR-spektroskopialla.

TULOKSET: Rasvaisen kalan kulutuksen lisääminen suurensi suurten HDL-lipoproteiinipartikkeleiden ja näiden lipidikomponenttien seerumipitoisuutta.

PÄÄTELMÄT: Rasvaisen kalan syönnillä on suotuisia vaikutuksia HDL-lipoproteiinien aineenvaihduntaan, sillä se lisää suurten HDL-lipoproteiinipartikkelien ja niiden sisältämien lipidien seerumipitoisuutta. Suurikokoiset HDL-partikkelit on yhdistetty pienentyneeseen sydän- ja verisuonitautiriskiin.

Kalan rasva sisältää runsaasti omega-3-sarjan monityydyttymättömiä rasvahappoja kuten eikosapentaeenihappoa (EPA) ja dokosaheksaeenihappoa (DHA). Rasvaisen kalan syönnillä ja monityydyttymättömillä rasvahapoilla on osoitettu olevan sydäntautiriskiä ja -kuolleisuutta pienentävä vaikutus (Studer ym. 2005, He 2009, Chiuve ym. 2012, Mozaffarian ym. 2012). Mekanismit, joilla kalan rasva suojaa valtimotaudeilta, eivät ole täysin selvillä. Lipoproteiiniaineenvaihdunnalla on ajateltu olevan keskeinen suojaava rooli (Balk ym. 2006, Voight ja Peloso ym. 2012).

Sydän- ja verisuonitautien keskeisiä riskitekijöitä ovat seerumin suurentunut LDL-pitoisuus ja pienentynyt HDL-pitoisuus, ja etenkin LDL- ja HDL-kolesterolipitoisuuksien suhdetta pidetään hyvänä suurentuneen riskin arvioinnissa. Suuri LDL-kolesterolipitoisuus on yhteydessä valtimonkovettumistautiin, mutta HDL-kolesterolin rooli on jäänyt epäselväksi, vaikka pienentynyt HDL-kolesterolipitoisuus on liitetty lisääntyneeseen sepelvaltimotautiriskiin (Besler ym. 2012, Voight ja Peloso ym. 2012).

Tuoreet geneettiset tutkimukset eivät ole osoittaneet HDL-partikkeleiden suoraa yhteyttä ateroskleroosiin (Deloukas ym. 2012). HDL-partikkelit ovat kooltaan ja tehtäviltään hyvin heterogeeninen joukko erilaisia partikkeleita, joten niiden tarkempi jaottelu eri alaluokkiin voi auttaa paremmin ymmärtämään HDL:n merkitystä sairauksien synnyssä (Pirillo 2013). Onkin osoitettu, että suurikokoiset HDL-partikkelit ovat käänteisessä yhteydessä sydän- ja verisuonitautien kehittymiseen, kun taas pienikokoiset HDL-partikkelit saattavat jopa lisätä sairastumisriskiä (Nofer ym. 2001, Arsenault 2009, Asztalos ym. 2011, Pirillo 2013). HDL-partikkelien antiaterogeeninen vaikutus perustunee kolmeen ominaisuuteen: Ne puhdistavat valtimoita vastaanottamalla kolesterolia sisäkalvon (intima) makrofageilta, ja suurikokoiset runsaasti kolesterolia sisältävät HDL-partikkelit kuljettavat kolesterolin maksaan metaboloitavaksi. Lisäksi HDL-partikkelit ja niiden komponentit voivat toimia

i+ = Artikkeliin liittyy internetoheisaineistoa

Duodecim 2013;129:2661–70

antioksidantteina ja ehkäistä valtimoseinämän tulehduksen syntymistä.

Omega-3-sarjan pitkäketjuisten monityydyttymättömien rasvahappojen yhteyttä HDL-partikkelien alaluokkiin on tutkittu, mutta suurin osa tutkimuksista on tehty kalan sijaan kalaöljyvalmisteilla (TAULUKKO 1). EPA:lla ja DHA:lla on havaittu olevan isoja HDL-partikkeleita lisäävä tai pieniä HDL-partikkeleita vähentävää vaikutus tai molemmat (Burillo 2012).

Lipoproteiinipartikkeleiden kokoa ja niiden sisältämien lipidien pitoisuuksia voidaan tutkia ^{1}H-NMR-spektroskopialla, jolla pystytään määrittämään kvantitatiivisesti eri HDL-alaluokkien seerumipitoisuudet ja keskimääräinen HDL-partikkelikoko (Ala-Korpela 2008, Mora ym. 2009).

Aineisto ja menetelmät

Tutkimukseen osallistui 131 iältään 40–70-vuotiasta naista ja miestä. Heidät satunnaistettiin kolmeen ryhmään, jotka olivat kaladieetti-, täysjyvä- ja verrokkiryhmä. Tutkimus kesti 12 viikkoa, ja loppuun asti mukana pysyi 105 henkilöä, joille tehtiin lipidianalyysi (kaladieetti n = 37, täysjyvä n = 34 ja verrokkiryhmä n = 35). Tutkimuksen sisäänottokriteereinä olivat plasman suurentunut paastoglukoosipitoisuus (5,6–6,9 mmol/l) tai heikentynyt glukoosinsieto (oraalisessa glukoosirasituskokeessa kahden tunnin plasman glukoosipitoisuus 7,8–11,0 mmol/l) ja vähintään kaksi seuraavista metabolisen oireyhtymän piirteistä: ylipaino tai lihavuus (painoindeksi 26–39 kg/m^2), keskivartalolihavuus (vyötärön-

TAULUKKO 1. Aikaisemmat tutkimukset kalan rasvan sekä omega-3:n, EPA:n ja DHA:n lisäämisen vaikutuksesta HDL-partikkeleihin (mukailtu: Burillo 2012).

Tutkimus (vuosi)	Tutkimuksen kohdehenkilöt	N	Tutkimus	EPA:n ja DHA:n saanti (g/vrk)	Kesto (vko)	Vaikutus HDL-lipoproteiineihin
Suzukawa (1995)	Hypertensiiviset	20	Kalaöljy	4	6	Ei vaikutusta
Dunstan (1997)	Tyypin 2 diabeetikot, joilla TG↑ ja/tai HDL-C↓	49	Kalaa kerran vuorokaudessa, liikunta ja vähärasvainen ruokavalio	3,6	8	HDL$_2$[1]↑, HDL$_3$[2]↓
Mori (2000)	Ylipainoiset, lievästi hyperkolesterolemiset miehet	56	EPA-, DHA-lisät	4	6	HDL$_2$↑, HDL$_3$↓
Woodman (2002)	Tyypin 2 diabeetikot, joilla hypertensio	51	EPA- ja DHA-lisät	4	6	HDL$_2$↑, HDL$_3$↓
Tholstrup (2004)	Terveet miehet	16	Kalaöljy	3	3	HDL$_2$↑
Wilkinson (2005)	Miehet, joilla TG↑ ja HDL-C↓	57	EPA- ja DHA-lisä	3	12	HDL$_2$↑
Griffin (2006)	Terveet henkilöt	258	Rasvainen kala; rasvahapposaannin omega-6 : omega-3-suhde ↓	1	27	HDL$_2$↑
Caslake (2008)	Terveet henkilöt	312	EPA- ja DHA-lisä	0,7–1,8	8	HDL$_2$↑, HDL$_3$↓
Lindqvist (2009)	Ylipainoiset miehet	35	Rasvainen kala viidesti viikossa	1,2	6	HDL$_2$↑
Maki (2011)	Hyperkolesterolemiset	31	omega-3-lisä	4	6	HDL-C↑, HDL-partikkelikoko↑

[1]HDL$_2$ = suurikokoisia HDL-partikkeleita, [2]HDL$_3$ = pienikokoisia HDL-partikkeleita

ympärys miehillä > 102 cm, naisilla > 88 cm), hypertriglyseridemia (seerumin triglyseridipitoisuus > 1,7 mmol/l), pieni seerumin HDL-kolesterolipitoisuus (miehillä < 1,0, naisilla < 1,3) ja kohonnut verenpaine (systolinen ≥ 130 mmHg, diastolinen 85 mmHg) (NCEP Adult Treatment Panel III 2001).

Antropologiset mittaukset suoritettiin tutkimuksen alussa ja lopussa (Lankinen ym. 2011, de Mello ym. 2011). Tutkimushenkilöistä 86 %:lla oli jokin pitkäaikainen lääkitys; eniten käytettyjä lääkkeitä olivat statiinit, ACE:n estäjät, ATR-salpaajat, beetasalpaajat, kalsiumin vastavaikuttajat ja antikoagulantit. Suurimmalla osalla lääkitys säilyi muuttumattomana koko tutkimuksen ajan. Rasva-aineenvaihduntaan vaikuttavien lääkkeiden käytössä ei ollut interventioryhmien eikä kalankäyttötertiilien välillä eroa.

Ruokavaliointerventio

Kaladieettiryhmän (n = 37) henkilöitä ohjattiin syömään rasvaista kalaa ja kalavalmisteita kolme kertaa viikossa (100–150 g kalaa/annos). Vähärasvaisia kaloja ei suositeltu käytettäväksi tutkimuksen aikana. Kalan valmistuksessa henkilöitä ohjeistettiin välttämään tyydyttyneen rasvan lähteitä kuten voita, voipohjaisia rasvoja ja kermaa.

Täysjyväryhmässä (n = 34) kalan käytön suhteen ei tehty muutoksia, ja verrokkiryhmän (n = 35) kalan syöntiä rajoitettiin kertaan viikossa. Ruokavaliointerventio sisälsi muutoksia lisäksi viljatuotteiden ja mustikoiden käytössä (KUVA 1). Tutkimushenkilöitä ohjeistettiin säilyttämään muut elintapansa ennallaan. Avainelintarvikkeet joko korvattiin tutkittaville, tai ne luovutettiin heille tutkimusyksiköstä (Lankinen ym. 2011).

Ruokavalion seuranta

Tutkimushenkilöt täyttivät ruokapäiväkirjan kerran ennen tutkimuksen alkua (viikko 0) ja kolmesti tutkimusruokavalion aikana: 3., 7. ja 11. viikolla. Ruokapäiväkirjaan kirjattiin ennalta määrättyjen neljän peräkkäisen päivän ajalta kaikki nautitut elintarvikkeet annos- tai grammamäärineen. Ruokapäiväkirjat analysoitiin Nutrica-ravintoainelaskentaohjelmalla (v. 3.1). Yksityiskohtaiset kalan käyttöä koskevat tiedot poimittiin erikseen ruokapäiväkirjoista.

Intervention aikaisen kalan kokonaiskulutuksen laskemiseksi Kaladieettiryhmässä täytettiin myös käyttökysely kalan kulutuksesta. Tähän merkittiin tukkimiehen kirjanpidolla kunakin päivänä nautittujen kala-annosten lukumäärä.

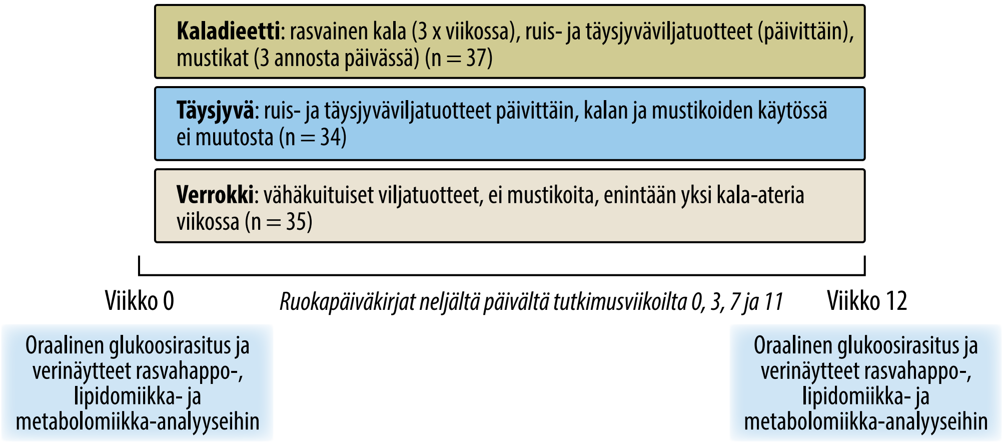

KUVA 1. Tutkimusasetelma ja ruokavaliot.

Biokemialliset analyysit

Verinäytteet otettiin ennen ja jälkeen intervention aamulla vähintään 12-tuntisen paaston jälkeen.

Seerumin lipidit. Seerumin kokonais-, LDL- ja HDL-kolesteroli- sekä kokonaistriglyseridipitoisuudet analysoitiin käyttäen kaupallisia reagensseja ja Thermo Fisher Konelab 20XT -analysaattoria (Thermo Electron Corporation; reagenssit 981813, 981656, 981823 ja 981786).

Glukoosi ja insuliini. Plasman insuliinipitoisuus analysoitiin kemiluminesenssi-immunomääritystä käyttäen ja plasman glukoosipitoisuus määritettiin glukoosi-heksokinaasimenetelmällä.

Lipoproteiinialaluokkien ja seerumin rasvahappokoostumuksen määrittäminen. Lipoproteiinialaluokkien pitoisuudet määritettiin seeruminäytteistä ^{1}HNMR-spektroskopiaan (proton nuclear magnetic resonance) perustuvalla menetelmällä (Ala-Korpela 2008, Soininen ym. 2009). Menetelmä mahdollistaa 14 lipoproteiinipartikkelialaluokan analysoinnin. HDL-partikkelialaluokat ovat erittäin suuri (XL), suuri (L), keskikoinen (M) ja pieni (S). Niiden keskimääräiset halkaisijat ovat 14,3 nm, 12,1 nm, 10,9 nm ja 8,7 nm. Keskimääräinen VLDL-, LDL- ja HDL-partikkelikoko lasketaan kyseisten alaluokkien partikkelikonsentraatioilla painotettuna keskiarvona. Menetelmän resoluution mahdollistamissa rajoissa kustakin lipoproteiinialaluokasta määritetään myös niiden päälipidikategorioiden (fosfolipidit, triglyseridit sekä vapaa ja esteröitynyt kolesteroli) pitoisuus seerumissa. Menetelmän yksityiskohdat on julkaistu aiemmin (Soininen ym. 2009, Inouye ym. 2010). EPA:n ja DHA:n plasmapitoisuudet analysoitiin kaasukromatografialla (Lankinen ym. 2011).

Tilastolliset menetelmät

Aineiston tilastollinen käsittely tehtiin SPSS- ja R-ohjelmilla (versiot 14.0 ja 2.7.2.). Muuttujien normaalisuudet arvioitiin histogrammijakaumasta tai Kolmogorov–Smirnovin testillä Lillieforsin korjauskerrointa käyttäen. Ryhmien väliset erot tutkimuksen alussa ja lopussa (ajan ja ryhmän interaktio) analysoitiin lineaarisella sekamallilla (linear mixed model), jossa huomioitiin mahdollisina sekoittavina tekijöinä tutkittavien ikä, sukupuoli, verenglukoosipitoisuuden paastoarvo, painoindeksi, statiini- ja diureesilääkitys sekä tupakointi. Raportoidut FDR-p-arvot (false discovery rate) huomioivat monivertailun. Kalankäytön yhteydet seerumin lipideihin ja lipoproteiineihin analysoitiin käyttäen Spearmanin järjestyskorrelaatioanalyysiä. Korrelaatiot analysoitiin myös osittaiskorrelaatioanalyysillä (partial correlation), jossa huomioitiin tutkittavien painoindeksi, insuliiniherkkyysindeksi (HOMA-indeksi), statiinien käyttö ja muutokset kuidun saannissa. Vakioinnit eivät muuttaneet tuloksia. Testasimme myös korrelaation HDL-partikkelikoon ja seerumin triglyseridipitoisuuksien välillä. Jatkoanalyyseissä tutkimushenkilöt jaettiin tertiileihin kalan käytössä tapahtuneiden muutosten perusteella, ja HDL-partikkeleissa tapahtuneita muutoksia vertailtiin näissä luokissa käyttämällä yksisuuntaista ANOVAa ja Bonferronin testiä.

Tulokset

Tutkimushenkilöiden kliiniset tiedot. Ryhmien välillä ei tutkimuksen alussa ollut merkitseviä eroja kliinisissä muuttujissa (TAULUKKO 2), eikä tutkimuksen kuluessa niissä todettu tapahtuneen merkitseviä muutoksia. Eri ruokavalioiden vaikutukset glukoosiaineenvaihduntaan, seerumista mitattuihin tulehdustekijöihin ja valtimon sisäpinnan toimintaan on raportoitu toisaalla (Lankinen ym. 2011, de Mello ym. 2011).

Ruokavalio. Kaladieettiryhmällä kalan kokonaiskulutus aloitusviikosta (viikko 0) tutkimusjakson loppuun (12. viikko) lähes kaksinkertaistui (TAULUKKO 3). Täysjyväryhmällä kalan kulutus säilyi suunnilleen samana, ja verrokkiryhmällä kulutus väheni, mutta muutos ei ollut tilastollisesti merkitsevä.

Tutkimusjakson aikana kaladieettiryhmäläiset nauttivat kalaa keskimäärin 3,3 annos-

TAULUKKO 2. Tutkimushenkilöiden kliiniset tiedot (keskiarvo ± keskihajonta).

	Kaladieetti (n = 37)	Täysjyvä (n = 34)	Verrokki (n = 35)
**Sukupuoli (mies/nainen)**	17/20	17/17	18/17
**Ikä (v)**	58 ± 7	58 ± 8	59 ± 7
**Painoindeksi (kg/m²)**	31,1 ± 3,6	31,4 ± 3,4	31,0 ± 3,6
**Vyötärönympärys (cm)**	106 ± 10	106 ± 11	106 ± 10
**Plasman glukoosipitoisuuden paastoarvo (mmol/l)**	6,1 ± 0,5	6,1 ± 0,4	6,2 ± 0,5
**Plasman glukoosipitoisuus (mmol/l) (OGTT, 2 tuntia)**	6,7 ± 1,7	6,6 ± 1,6	6,8 ± 1,9
**Seerumin insuliinipitoisuus (mU/l)**	11,7 ± 5,9	12,0 ± 6,2	13,0 ± 6,7
**Systolinen verenpaine (mmHg)**	137 ± 13	135 ± 16	139 ± 12
**Diastolinen verenpaine (mmHg)**	89 ± 7	86 ± 8	88 ± 7
**Seerumin kolesterolipitoisuus (mmol/l)**	5,1 ± 0,9	5,1 ± 1,0	5,4 ± 1,0
**LDL-kolesterolipitoisuus (mmol/l)**	3,1 ± 0,7	3,2 ± 0,8	3,4 ± 0,8
**HDL kolesterolipitoisuus (mmol/l)**	1,3 ± 0,3	1,2 ± 0,4	1,3 ± 0,3
**Seerumin triglyseridipitoisuus (mmol/l)**	1,6 ± 0,6	1,5 ± 0,8	1,5 ± 0,8

TAULUKKO 3. Kalankulutus sekä EPA:n ja DHA:n saanti (g/päivä; keskiarvo ± keskihajonta) tutkimusryhmissä ruokapäiväkirjojen mukaan[1].

	Kaladieetti (n = 37)		Täysjyvä (n = 34)		Verrokki (n = 35)	
**Kalalaji**	**0 vko**	**Intervention aikana**	**0 vko**	**Intervention aikana**	**0 vko**	**Intervention aikana**
Lohi	18 ± 31	22 ± 22	12 ± 22	10 ± 18	12 ±27	6,5 ± 9,3
Muikku	0,8 ± 4,9	7,1 ± 13*	1,4 ± 8,45	2,9 ± 13	4,5 ± 18	1,1 ± 4,4
Kaikki kalat	36 ± 39	67 ± 32*	37 ± 35	42 ± 46	28 ± 39	16 ± 15
EPA	0,15 ± 0,13	0,22 ± 0,09*	0,14 ± 0,24	0,12 ± 0,13	0,10 ± 0,10	0,06 ± 0,05*
DHA	0,35 ± 0,28	0,59 ± 0,27*	0,30 ± 0,35	0,31 ± 0,29	0,22 ± 0,24	0,17 ± 0,13*

[1]0-viikolla 1 x 4 vrk ja intervention aikana 3 x 4 vrk
*Ryhmän sisäinen ero, p-arvo < 0,05

ta (100–150 g /annos) viikossa. Rasvaisen kalan määrä oli määrästä peräti 91 %. Kaladieettiryhmässä tyydyttyneiden rasvahappojen saanti pieneni, kun taas EPA:n, DHA:n ja alfalinoleenihapon (ALA) saanti suureni. Täysjyväryhmässä rasvansaanti kokonaisuudessaan pieneni, ja verrokkiryhmässä monityydyttymättömien rasvahappojen (mukaan lukien EPA ja DHA) saanti pieneni (Lankinen ym. 2011).

Seerumin HDL-partikkelien ja rasvahappojen muutokset. Lipoproteiinipartikkelien alaluokissa ja niiden lipidipitoisuuksissa ei tapahtunut merkitseviä muutoksia, kun niitä tarkasteltiin ajan ja ryhmän yhteisvaikutuksena (INTERNETOHEISAINEISTON TAULUKKO). Ryhmän sisäisissä vertailuissa kaladieettiryhmässä oli kuitenkin havaittavissa suuntaus HDL-partikkeleiden keskimääräisen halkaisijan suurenemiseen sekä suurten ja erittäin suurten HDL-partikkeleiden suureneviin seerumipitoisuuksiin (INTERNETOHEISAINEISTON TAULUKKO). Suurten HDL-partikkeleiden lipideissä näkyi myös suuntaus seerumipitoisuuksien suurenemiseen kaladieettiryhmässä. HDL-partikkelien keskimääräinen läpimitta oli negatiivisesti yhteydessä seerumin triglyseridipitoisuuden (tutkimuksen alussa:

r-arvo = – 0,285, p = 0,004; tutkimuksen lopussa: r-arvo = –0,346, p = 3,4 x 10^{-4}).

Monityydyttymättömien omega-3 rasvahappojen, EPA:n ja DHA:n pitoisuudet suurenivat kaladieettiryhmässä merkitsevästi (INTERNETOHEISAINEISTON TAULUKKO). Merkittävimmät partikkelikoossa tapahtuneet muutokset ja eniten muuttuneet lipidipitoisuudet valittiin lähempään tarkasteluun (KUVA 2).

Kalan laadun ja määrän vaikutukset HDL-partikkeleihin koko aineistossa. Kalan kulutuksen lisääminen oli yhteydessä HDL-partikkelien läpimitan suurenemiseen (r-arvo = 0,333, p-arvo = 0,001) ja isojen HDL-partikkelien pitoisuuden nousuun (r-arvo = 0,356, p-arvo = 2,6 x 10^{-4}). Myös suurten HDL-partikkeleiden eri lipidiluokkien seerumipitoisuudet olivat yhteydessä kalan käytössä tapahtuviin muutoksiin (r-arvo = 0,306–0,391, p-arvo = 0,001–0,030). Vakiointi painoindeksillä, insuliiniherkkyysindeksillä ja kuidun saannilla ei muuttanut tuloksia. Suurten HDL-partikkeleiden pitoisuuksissa tapahtuneet muutokset korreloivat merkitsevästi (p-arvo < 0,05) myös seerumista mitattujen EPA- ja DHA-pitoisuusmuutosten kanssa (TAULUKKO 4).

Koko tutkimusaineisto jaettiin edelleen kalan kulutuksessa tapahtuneiden muutosten perusteella kolmeen osaan: 1. kolmanneksella rasvaisen kalan kulutus väheni, 2. kolmanneksella se pysyi suurin piirtein samana ja 3. kolmanneksella se lisääntyi tutkimusjakson aikana. Samoin tapahtui myös plasman EPA- ja DHA-pitoisuuksille. Kahden ensimmäisen kolmanneksen henkilöt kuuluivat pääosin täysjyvä- ja verrokkiryhmiin ja kolmannen kolmanneksen henkilöt pääosin kaladieettiryhmään, mutta jokaisessa kolmanneksessa oli henkilöitä kaikista dieettiryhmistä. KUVASSA 2 on esitetty yhteenveto keskeisimmistä HDL-aineenvaihduntaa kuvaavista muutoksista. Suurten HDL-partikkeleiden ja niiden lipidikomponenttien seerumipitoisuus suureni huomattavasti kalan käytön lisääntyessä (3. kolmannes), kun ensimmäisessä ja toisessa kolmanneksessa muutokset olivat vähäisiä.

Pohdinta ja päätelmät

Lisääntyneellä rasvaisen kalan kulutuksella todettiin merkitsevä yhteys pitkäketjuisten monityydyttymättömien omega-3 rasvahappojen (EPA ja DHA) plasmapitoisuuksien, HDL-partikkelien keskimääräisen läpimitan, suurten HDL-partikkelien pitoisuuden ja suurten HDL-partikkelien lipidien pitoisuuksien suurenemiseen. Tämä on merkittävä havainto, koska suurikokoisten HDL-partikkelien tiedetään olevan käänteisessä yhteydessä sydän- ja verisuonitautien kehittymiseen,

TAULUKKO 4. Plasman EPA- ja DHA -pitoisuuksissa tapahtuneiden muutosten korrelaatiot suurten HDL-partikkeleiden keskeisiin muutoksiin koko tutkimusjoukossa.

HDL-partikkeli (muutos 0–12 viikolla)	EPA (n = 105) muutos 0–12 viikolla		DHA (n = 105) muutos 0–12 viikolla	
	r-arvo	p-arvo	r-arvo	p-arvo
XL HDL P	0,428	< 0,0001	0,468	< 0,0001
L HDL P	0,333	0,001	0,254	0,011
HDL D	0,398	< 0,0001	0,377	< 0,0001
L HDL L	0,328	0,001	0,273	0,006
L HDL PL	0,284	0,004	NS	NS
L HDL C	0,31	0,002	0,312	0,002
L HDL CE	0,27	0,007	0,275	0,006
L HDL FC	0,372	< 0,0001	0,368	< 0,0001
XL HDL TG	NS	NS	NS	NS

XL = erittäin suurikokoinen, L = suurikokoinen, P = partikkelikonsentraatio, D = halkaisija, L = kokonaislipidipitoisuus, PL= fosfolipidit, C = kolesteroli, CE = kolesteroliesterit, FC = vapaa kolesteroli, TG = triglyseridit

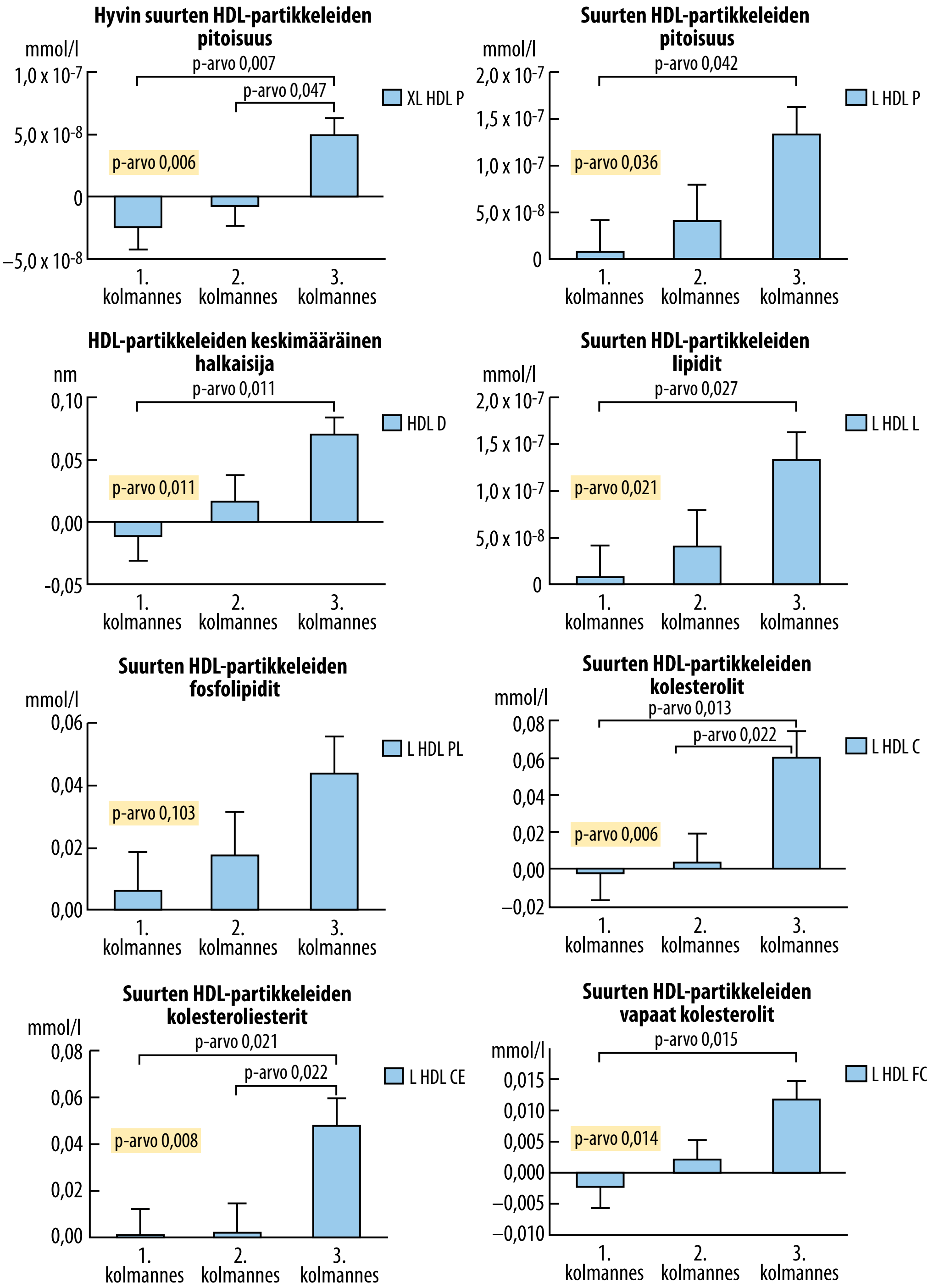

KUVA 2. HDL-partikkeleiden koossa ja lipidikategorioissa tapahtuneet muutokset eri kalankulutuskolmanneksissa. Tutkittavat on jaettu kolmanneksiin kalankäytössä tapahtuneiden muutosten perusteella. Kalankäyttö on määritetty ruokapäiväkirjoista. Laatikoitu p-arvo kuvastaa vertailua kaikkien kolmannesten välillä (yksisuuntainen ANOVA-testi).

YDINASIAT

- Rasvaisella kalalla ja sen sisältämillä omega-3-sarjan monityydyttymättömillä rasvahapoilla on havaittu väestötutkimuksissa ja interventiotutkimuksissa sydän- ja verisuonitautiriskiä pienentävä vaikutus.
- HDL-partikkeleiden kokoa ja lipidejä voidaan tutkia ^{1}HNMR-spektroskopialla.
- Runsaasti rasvaista kalaa käyttäneillä HDL-partikkelien keskimääräinen koko ja suurten HDL-partikkelialaluokkien pitoisuudet seerumissa suurenivat.
- Suurikokoiset HDL-partikkelit on liitetty pienentyneeseen sydän- ja verisuonitautiriskiin.

mikä liittyy mahdollisesti tehostuneeseen kolesterolin takaisin kuljetukseen (Pirillo 2013).

Rasvaisen kalan käytöllä ei ollut merkitsevää vaikutusta seerumin HDL-kolesterolin kokonaispitoisuuteen. Myöskään kokonaiskolesterolin, LDL-kolesterolin ja triglyseridien pitoisuudet eivät muuttuneet. Aiemmin olemme raportoineet, että kyseinen rasvaista kalaa, täysjyväviljaa ja mustikoita sisältänyt ruokavalio paransi glukoosiaineenvaihduntaa sekä endoteelin toimintaa ja tulehdusta (Lankinen ym. 2011, De Mello ym. 2011). Parantunut glukoosiaineenvaihdunta näytti olevan yhteydessä erityisesti plasman suurentuneisiin EPA- ja DHA-pitoisuuksiin (Lankinen ym. 2011). Huolimatta siitä, että muutoksia HDL-kolesterolin kokonaispitoisuuksissa ei havaittu, kalan kulutuksen lisääminen oli yhteydessä HDL-partikkelien keskimääräisen läpimitan suurentumiseen sekä suurten ja erittäin suurten HDL-partikkelien pitoisuuksien lisääntymiseen (KUVA 2). Myös suurten HDL-partikkelien lipidipitoisuudet kasvoivat rasvaisen kalan käytön lisääntymisen myötä, mikä lienee ensisijaisesti seurausta suurten HDL-partikkelien määrän lisääntymisestä, mutta myös koostumuksessa on saattanut tapahtua muutoksia. Havainnot säilyvät samana, kun tarkastellaan kalan käytön sijaan muutoksia plasman EPA- ja DHA-pitoisuuksissa. Tässä tutkimuksessa havaittiin myös HDL:n keskimääräisen läpimitan ja seerumin triglyseridien käänteinen yhteys.

Suurten HDL-partikkelien pitoisuuden lisääntyminen saattaa liittyä kolesterolin takaisinkuljetuksen kiihtymiseen (Khera ym. 2011). Kolesterolin käänteiskuljetuksella voi olla merkittävä rooli ateroskleroosin ehkäisyssä, ja HDL-alaluokat kuvastavat käänteiskuljetuksen tehokkuutta paremmin kuin HDL:n kokonaispitoisuus (von Eckardstein ym. 2001). Myös aiemmissa tutkimuksissa on havaittu, että kalaöljy lisää nimenomaan suurten HDL-partikkelien pitoisuuksia (TAULUKKO 1). On myös näyttöä siitä, että kalaöljy lisää kolesterolin takaisinkuljetusta makrofageista ja sen erittymistä maksan kautta sappeen (Nishimoto ym. 2009). Kalaöljyn käytön yhteys sydän- ja verisuonitauteihin ei ole kuitenkaan selvä. Tuoreessa meta-analyysissa, joka perustui 20 tutkimukseen, omega-3-sarjan monityydyttymättömien rasvahappojen lisäämisellä ei todettu olevan merkitsevää vaikutusta sydän- ja verisuonitautien kuolemanriskiin (Rizos ym. 2012). On syytä todeta, että tämä kalaöljyvalmisteisiin liittyviä meta-analyysi ei kumoa rasvaisen kalan merkitystä terveyden edistäjänä, sillä kala sisältää rasvan lisäksi myös muita terveydelle edullisia ainesosia kuten esimerkiksi hyvälaatuista proteiinia.

Tutkimushenkilöt jaettiin kolmanneksiin sen perusteella, kuinka paljon heidän kalansyöntinsä tutkimuksen aikana muuttui. Kolmannessa tertiilissä, jossa kalankäyttö lisääntyi eniten, havaittiin myös suurimmat muutokset HDL-partikkeleissa. Tässä ryhmässä kalankäyttö oli keskimäärin 74 g vuorokaudessa eli yli puoli kiloa viikossa. Suurin osa kalasta oli rasvaista kalaa. Tämä poikkeaa suomalaisten keskimääräisestä kalankäytöstä merkittävästi, sillä 25–64-vuotiaat miehet syövät kalaa vain 28 g/vrk ja naiset 24 g/vrk. Vanhemmilla (65–74-vuotiaat) käyttö on hieman runsaampaa, sillä tässä ikäryhmässä miehet syövät kalaa keskimäärin 40 g/vrk ja naiset 32 g/vrk (Paturi ym. 2008).

Tutkimuksen heikkous on se, että varsinaiset päätulokset liittyvät niin sanottuihin

sekundaarianalyyseihin, sillä varsinaisten tutkimusruokavalioiden vaikutuksesta emme nähneet tilastollisesti merkitseviä muutoksia suhteessa verrokkiruokavalioon. Näyttää kuitenkin vahvasti siltä, että juuri kalan käytöllä on itsenäinen vaikutus HDL-aineenvaihduntaan, sillä kalankäytön muutosten mukaan jaetut kolmannekset sisälsivät henkilöitä kaikista interventioryhmistä, joten esimerkiksi täysjyväviljan tai mustikoiden käytön lisääminen ei siis selittänyt näitä tuloksia. Myöskään vakiointi kuidunsaannilla ei muuttanut tuloksia.

Pitkäketjuisilla omega-3-sarjan monityydyttymättömillä rasvahapoilla on todettu sydän- ja verisuonitaudeilta suojaavia vaikutuksia. Suurikokoiset HDL-partikkelit on liitetty pienentyneeseen valtimonkovettumistaudin riskiin. Tässä tutkimuksessa rasvaisen kalan syönnin lisääminen oli yhteydessä seerumin HDL-partikkelien keskimääräisen koon suurenemiseen sekä suurten HDL-partikkelien ja niiden lipidien pitoisuuksien suurenemiseen. Tutkimus viittaa siihen, että rasvainen kala saattaa vaikuttaa valtimonkovettumistaudin riskiin muuttamalla HDL:n aineenvaihduntaa suuntaan, johon liittyy pienentynyt sairastumisriski ja kuolleisuus. Tulokset vahvistavat aikaisempaa näyttöä siitä, että kalankäyttöä kannattaa suositella henkilöille, joilla on heikentynyt glukoosiaineenvaihdunta tai suurentunut sydän- ja verisuonitaudin riski. Sen sijaan kalaöljyvalmisteiden käyttöön ei tämän tutkimuksen perusteella voida ottaa kantaa. ■

LAURA JOUKAMO, LK
JUSSI PAANANEN, FT
HANNU MYKKÄNEN, professori
Itä-Suomen yliopisto, lääketieteen laitos, kansanterveystieteen ja kliinisen ravitsemustieteen yksikkö

MARIA LANKINEN, FT, tutkijatohtori
MARJUKKA KOLEHMAINEN, FT, dosentti
Itä-Suomen yliopisto, lääketieteen laitos, kansanterveystieteen ja kliinisen ravitsemustieteen yksikkö
Teknologian tutkimuskeskus VTT, Kuopio

URSULA SCHWAB, FT, apulaisprofessori
Itä-Suomen yliopisto, lääketieteen laitos, kansanterveystieteen ja kliinisen ravitsemustieteen yksikkö
KYS, medisiininen keskus, kliinisen ravitsemuksen yksikkö

KAISA POUTANEN, akatemiaprofessori
Itä-Suomen yliopisto, lääketieteen laitos, kansanterveystieteen ja kliinisen ravitsemustieteen yksikkö
Teknologian tutkimuskeskus VTT, Espoo

HELENA GYLLING, LKT, emeritaprofessori, sisätautien erikoislääkäri
HYKS, medisiininen tulosyksikkö, yleissisätaudit ja Biomedicum Helsinki

MATTI UUSITUPA, LKT, professori, sisätautien erikoislääkäri
Itä-Suomen yliopisto, lääketieteen laitos, kansanterveystieteen ja kliinisen ravitsemustieteen yksikkö
KYS, tutkimusyksikkö

TUULIKKI SEPPÄNEN-LAAKSO, FaT, dosentti, erikoistutkija

MATEJ OREŠIČ, tutkimusprofessori
Teknologian tutkimuskeskus VTT, Espoo

PASI SOININEN, FT
Oulun yliopisto, terveystieteiden laitos, laskennallinen lääketiede
Itä-Suomen yliopisto, farmasian laitos, NMR-metabolomiikkalaboratorio

ANTTI J. KANGAS, DI
Oulun yliopisto, terveystieteiden laitos, laskennallinen lääketiede

MIKA ALA-KORPELA, professori
Oulun yliopisto, terveystieteiden laitos, laskennallinen lääketiede
Itä-Suomen yliopisto, farmasian laitos, NMR-metabolomiikkalaboratorio
Bristolin yliopisto, laskennallinen lääketiede

SIDONNAISUUDET
Laura Joukamo: Ei sidonnaisuuksia
Jussi Paananen: Ei sidonnaisuuksia
Hannu Mykkänen: Asiantuntijapalkkio (EU), Matkakorvaus (EU)
Maria Lankinen: Ei sidonnaisuuksia
Marjukka Kolehmainen: Ei sidonnaisuuksia
Ursula Schwab: Ei sidonnaisuuksia
Kaisa Poutanen: Ei sidonnaisuuksia
Helena Gylling: Ei sidonnaisuuksia
Matti Uusitupa: Luentopalkkio (Professio, Elintarvikkeiden tutkimussäätiö)
Tuulikki Seppänen-Laakso: Ei ilmoitusta sidonnaisuuksista
Matej Orešič: Luentopalkkio (H. Lundbeck Ab)
Pasi Soininen: Ei sidonnaisuuksia
Antti J. Kangas: Ei sidonnaisuuksia
Mika Ala-Korpela: Ei sidonnaisuuksia

KIRJALLISUUTTA

- Ala-Korpela M. Critical evaluation of 1H NMR metabonomics of serum as a methodology for disease risk assessment and diagnostics. Clin Chem Lab Med 2008;46:27–42.
- Arsenault BJ, Lemieux I, Despres JP, ym. HDL particle size and the risk of coronary heart disease in apparently healthy men and women: the EPIC-Norfolk prospective population study. Atherosclerosis 2009;206:276–81.
- Asztalos BF, Tani M, Schaefer EJ. Metabolic and functional relevance of HDL subspecies. Curr Opin Lipidol 2011;22:176–85.
- Balk EM, Lichtenstein AH, Chung M, Kupelnick B, Chew P, Lau J. Effects of omega-3 fatty acids on serum markers of cardiovascular disease risk: a systematic review. Atherosclerosis 2006;189:19–36.
- Besler C, Lüscher TF, Landmesser U. Molecular mechanisms of vascular effects of High-density lipoprotein: alterations in cardiovascular disease. EMBO Mol Med 2012; 4:251–68.
- Caslake MJ, Miles EA, Kofler BM, ym. Effect of sex and genotype on cardiovascular biomarker response to fish oils: the FINGEN Study. Am J Clin Nutr 2008;88:618–29.
- Burillo E, Martin-Fuentes P, Mateo-Gallego R, ym. Omega-3 fatty acids and HDL. How do they work in the prevention of cardiovascular disease? Curr Vasc Pharmacol 2012;10:432–41.
- Chiuve E, Rimm EB, Roopinder KS, ym. Dietary fat quality and risk of sudden cardiac death in women. Clin Nutr 2012;96: 498–507.
- Deloukas P, Kanoni S, Willenberg C, ym. Large-scale association analysis identifies new risk loci for coronary artery disease. Nat Genet 2012;45:22–33.
- De Mello VD, Schwab U, Kolehmainen M, ym. A diet high in fatty fish, bilberries and wholegrain products improves markers of endothelial function and inflammation in individuals with impaired glucose metabolism in a randomised controlled trial: the Sysdimet study. Diabetologia 2011;54:2755–67.
- Dunstan DW, Mori TA, Puddey IB, ym. The independent and combined effects of aerobic exercise and dietary fish intake on serum lipids and glycemic control in NIDDM. Diabetes Care 1997;20:913–21.
- Griffin MD, Sanders TAB, Morgan LM, ym. Effects of alterin the ratio of dietary n-6 to n-3 fatty acids on insulin sensitivity, lipoprotein size, and postprandial lipemia in men and postmenopausal women aged 45–70 y: the OPTILIP Study. Am J Clin Nutr 2006;84:1290–8.
- He K. Fish, long-chain omega-3 polyunsaturated fatty acids and prevention of cardiovascular disease – eat fish or take fish oil supplement? Prog Cardiovasc Dis 2009; 52:95–114.
- Inouye M, Kettunen J, Soininen P, ym. Metabonomic, transcriptomic, and genomic cariation of a population cohort. Mol Syst Biol 2010;6:441.
- Khera AV, Cuchel M, de la Llera-Moya M, ym. Cholesterol efflux capacity, high-density lipoprotein function, and atherosclerosis. N Engl J Med 2011;364:127–35.
- Lankinen M, Schwab U, Kolehmainen M, ym. Whole grain products, fish and bilberries alter glucose and lipid metabolism in a randomized, controlled trial: the Sysdimet Study. PLoS ONE 2011;6:e22646.
- Lindqvist HM, Langkilde AM, Undeland I, Sandber AS. Herring (Clupea harengus) intake influences lipoproteins but not inflammatory and oxidation markers in overweight men. Brit Jour Nutr 2009;101:383–90.
- Maki KC, Lawless AL, Kelley KM, ym. Effects of prescription omega-3-acid ethyl esters on fasting lipid profile in subjects with primary hypercholesterolemia. J Cardiovasc Pharmacol 2011;57:489–94.
- Mora S, Otvos JD, Rifai N, Rosenson RS, Buring JE, Ridker PM. Lipoprotein particle profiles by Nuclear Magnetic Resonance compared with standard lipids and apolipoproteins in predicting incident cardio vascular disease in women. Circ 2009;119:931–9.
- Mori TA, Burke V, Puddey IB, ym. Purified eicosapentaenoic and docosahexaenoic acids have differential effects on serum lipids and lipoproteins, LDL particle size, glucose, and insulin in mildly hyperlipidemic men. Am J Clin Nutr 2000;71:1085–94.
- Mozaffarian D, Wu JH. (n-3) fatty acids and cardiovascular health: are effects of EPA and DHA shared or complementary? J Nutr 2012;142:614S–25S.
- Nishimoto T, Pellizzon MA, Aihara M, ym. Fish oil promotes macrophage reverse cholesterol transport in mice. Arterioscler Thromb Vasc Biol 2009;29:1502–8.
- Nofer JF, Levkau B, Junker R, ym. Suppression of endothelial cell apoptosis by high density lipoproteins (DHL) and HDL-associated lysosphingolipids. J Biol Chem 2001;276:34480–5.
- Paturi M, Tapanainen H, Reinivuo H, Pietinen P. The National FINDIET 2007 Survey. Publications of the National Public Health Institute, 2008, B 23.
- Pirillo A, Norata GD, Catapano AL. High-density lipoprotein subfractions – what the clinicians need to know. Cardiology 2013;124:116–125.
- Rizos EC, Ntzani EE, Bika E, Kostapanos MS, Elisaf MS. Association between omega-3 fatty acid supplementation and risk of major cardiovascular disease events, a systematic review and meta-analysis. JAMA 2012;308:1024–33.
- Soininen P, Kangas AJ, Würtz P, ym. High-throughput serum NMR metabonomics for cost-effective holistic studies on systemic metabolism. Analyst 2009;134:1781–5.
- Suzukawa M, Abbey M, Howe PRC, Nestel PJ. Effects of fish oil fatty acids on low density lipoprotein size, oxidazability, and uptake by macrophages. J Lipid Res 1995;36:473–84.
- Studer M, Matthias B, Leimenstoll B, Glass TR, Bucher HC. Effect of different antilipidemic agents and diets on mortality: a systematic review. Arch Intern Med 2005;165:725–9.
- Tholstrup T, Hellgren LI, Petersen M, ym. A solid dietary fat containing fish oil redistributes lipoprotein subclasses without increasing oxidative stress in men. J Nutr 2004;134:1051–7.
- Voight BF, Peloso G, Orho-Melander M, ym. Plasma HDL cholesterol and risk of myocardial infarction: a mendelian randomisation study. Lancet 2012;380:572–80.
- von Eckardstein A, Nofer J-R, Assmann G. High density lipoproteins and arteriosclerosis. Role of cholesterol effluz and reverse cholesterol transport. Arterioscler Thromb Vasc Biol 2001;21:13–27.
- Wilkinson P, Leach C, Ah-Sing EE, ym. Influence of a-linolenic acid and fish-oil on markers of cardiovascular risk in subjects with an atherogenic lipoprotein phenotype. Atherosclerosis 2005;181:115–24.
- Woodman RJ, Mori TA, Burke V, Puddey IB, Watts G, Beilin LJ. Effects of purified eicosapentaenoic and docosahexaenoic acids on glycemic control, blood pressure, and serum lipids in type 2 diabetic patients with treated hypertension. Am J Clin Nutr 2002;76:1007–15.

Summary

Fatty fish modifies HDL particle size and lipid concentrations

BACKGROUND: We investigated with ^{1}HNMR-spectroscopy the effects of habitual fatty fish intake on serum lipiprotein profiles in persons with features of metabolic syndrome.

MATERIAL AND METHODS: The participants (n = 105) were randomized into three diet intervention groups. The groups were given different dietary instructions.

RESULTS: Increased intake of fatty fish had a significant ($p < 0.05$) increasing effect on the amount of large HDL-lipoprotein subclasses and their lipids.

CONCLUSIONS: Frequent intake of fatty fish may have beneficial effects on HDL-metabolism beyond that assumed to be related to its serum concentrations.
