# Supplementary material for: Effects of Whole Grain, Fish and Bilberries on Serum Metabolic Profile and Lipid Transfer Protein Activities: A Randomized Trial (Sysdimet)
Source: PLoS One. 2014 Feb 28;9(2):e90352. doi: 10.1371/journal.pone.0090352 (PMC3938672; doi:10.1371/journal.pone.0090352)
Supplement: Method S1 — (DOCX) [file pone.0090352.s005.docx]

**Method S1**

**Plasma Glucose and serum insulin**

An oral glucose tolerance test (OGTT) was performed after a 12-h overnight fasting at the beginning and at the end of the intervention. In the OGTT the subjects ingested 75 g of glucose with 300 ml of water in maximum 5 min. Samples for plasma glucose and serum insulin analyses were drawn at 30, 60 and 120 min. Plasma glucose concentration was analyzed by using Konelab 20XTi Clinical Chemistry Analyzer and Enzymatic photometric (glucose hexokinase) method (Konelab System Reagents, Thermo Fisher Scientific, Vantaa, Finland). Serum insulin concentration was analyzed with a chemiluminescent immunoassay (Advia Centaur Immunoassay System, Siemens Medical Solution Diagnostics, Tarrytown, NY, USA).

### Serum cholesterol, Apo A-1 and ApoB100

Concentrations of serum total, LDL and HDL cholesterol and serum triglycerides were analyzed using commercial kits (981813, 981656, 981823 and 981786, respectively, Thermo Electron Corporation, Vantaa, Finland) and Thermo Fisher Konelab 20XTi Analyzer (Thermo Electron Corporation, Vantaa, Finland). Apo A-1 ja ApoB100 were analyzed by immunoturbimetry method (340 nm) using Konelab 20Xti Clinical Chemistry Analyzer (Thermo Fisher Scientific, Vantaa, Finland) and Konelab System Reagents Apolipoprotein A1 and Apolipoprotei B (Thermo Fisher Scientific, Finland).
